# Deep nasal sinus cavity microbiota dysbiosis in Parkinson’s disease

**DOI:** 10.1038/s41531-021-00254-y

**Published:** 2021-12-08

**Authors:** Gian Pal, Vivian Ramirez, Phillip A. Engen, Ankur Naqib, Christopher B. Forsyth, Stefan J. Green, Mahboobeh Mahdavinia, Pete S. Batra, Bobby A. Tajudeen, Ali Keshavarzian

**Affiliations:** 1 Department of Neurology, Rush University Medical Center, Chicago, IL USA; 2 Rush Medical College, Rush Center for Integrated Microbiome and Chronobiology Research, Rush University Medical Center, Chicago, IL USA; 3 Genomics and Microbiome Core Facility, Rush University Medical Center, Chicago, IL USA; 4 Department of Internal Medicine, Division of Infectious Diseases, Rush University Medical Center, Chicago, IL USA; 5 Department of Internal Medicine, Allergy/Immunology Division, Rush University Medical Center, Chicago, IL USA; 6 Department of Otorhinolaryngology—Head and Neck Surgery, Rush University Medical Center, Chicago, IL USA; 7 Department of Medicine & Physiology, Rush University Medical Center, Chicago, IL USA; 8 Division of Pharmacology, Utrecht Institute for Pharmaceutical Sciences, Utrecht University, Utrecht, Netherlands

**Keywords:** Olfactory system, Parkinson's disease

## Abstract

Olfactory dysfunction is a pre-motor symptom of Parkinson’s disease (PD) that appears years prior to diagnosis and can affect quality of life in PD. Changes in microbiota community in deep nasal cavity near the olfactory bulb may trigger the olfactory bulb-mediated neuroinflammatory cascade and eventual dopamine loss in PD. To determine if the deep nasal cavity microbiota of PD is significantly altered in comparison to healthy controls, we characterized the microbiota of the deep nasal cavity using 16S rRNA gene amplicon sequencing in PD subjects and compared it to that of spousal and non-spousal healthy controls. Correlations between microbial taxa and PD symptom severity were also explored. Olfactory microbial communities of PD individuals were more similar to those of their spousal controls than to non-household controls. In direct comparison of PD and spousal controls and of PD and non-spousal controls, significantly differently abundant taxa were identified, and this included increased relative abundance of putative opportunistic-pathobiont species such as *Moraxella catarrhalis*. *M. catarrhalis* was also significantly correlated with more severe motor scores in PD subjects. This proof-of-concept study provides evidence that potential pathobionts are detected in the olfactory bulb and that a subset of changes in the PD microbiota community could be a consequence of unique environmental factors associated with PD living. We hypothesize that an altered deep nasal microbiota, characterized by a putative pro-inflammatory microbial community, could trigger neuroinflammation in PD.

## Introduction

Parkinson’s disease (PD) is a complex neurodegenerative disease characterized by nigrostriatal degeneration resulting in bradykinesia, rigidity, tremor, and gait dysfunction^1^. Non-motor symptoms are also typically present, including depression, constipation, and alteration of smell. Diminished sense of smell (hyposmia), is a common hallmark of prodromal PD^2^.

Though PD’s etiology remains unknown, gut dysbiosis, or imbalance of the gut microbial community structure and function, has been implicated in disease pathogenesis. PD gut dysbiosis is characterized by increased putative pro-inflammatory microbes, belonging to the phylum Proteobacteria, and a reduction in putative beneficial short chain fatty acids (SCFAs)-producing bacteria (e.g., bacteria from the genera *Blautia*, *Roseburia*, and *Faecalibacterium*)^3^. This gut dysbiosis may ultimately contribute to systemic and neuroinflammation, possibly leading to alpha-synuclein misfolding and aggregation that is observed in PD intestinal and brain tissue^4,5^.

Given the loss of olfaction that has been associated with pre-motor PD in 75–95% of early cases^6^, the nasal cavity may be a secondary site (in addition to the gut) triggering neuroinflammation in PD and is hypothesized to serve as a route of pathogen invasion/toxin exposure, originally termed the “dual-hit hypothesis”^2,4,5^. Similar to the gut, the nasal cavity supports distinct microbial communities that inhabit the anterior nares, nasal vestibule, and middle meatus^7^. The rostral (deep) region of the sinusoidal cavity consists of a specialized epithelial layer proximate to the olfactory bulb^8^. This is inhabited by a stable microbial community that plays a role in olfactory development and the function of smell^7^. Changes in the microbiota composition of this region have been associated with a pro-inflammatory profile in diseases such as chronic rhinosinusitis, which is thought to reduce olfaction^9^.

Previous studies failed to find differences between PD and healthy subjects in the external nostril and nasal wash microbiome^10,11^. However, the biological linkage of the nostril or nasal wash microbiome in neuroinflammation is debatable. This study examines the PD microbiota community in the deep nasal sinus cavity proximal to the olfactory bulb. Rigorous sample collection for microbiota analysis was performed by rhinologists experienced in nasal sample collection after complete nasal endoscopy, using endoscopy guided small size nasal swabs from the middle meatus. We posit that the unexplored deep nasal sinus cavity is a more relevant site for neuroinflammation in PD and hypothesized that the deep nasal microbiota community has a pro-inflammatory profile in PD.

## Results

### Comparison of nasal microbiota between random non-household (rHC) and spousal household healthy controls (SpHC)

**Fig. 1:**
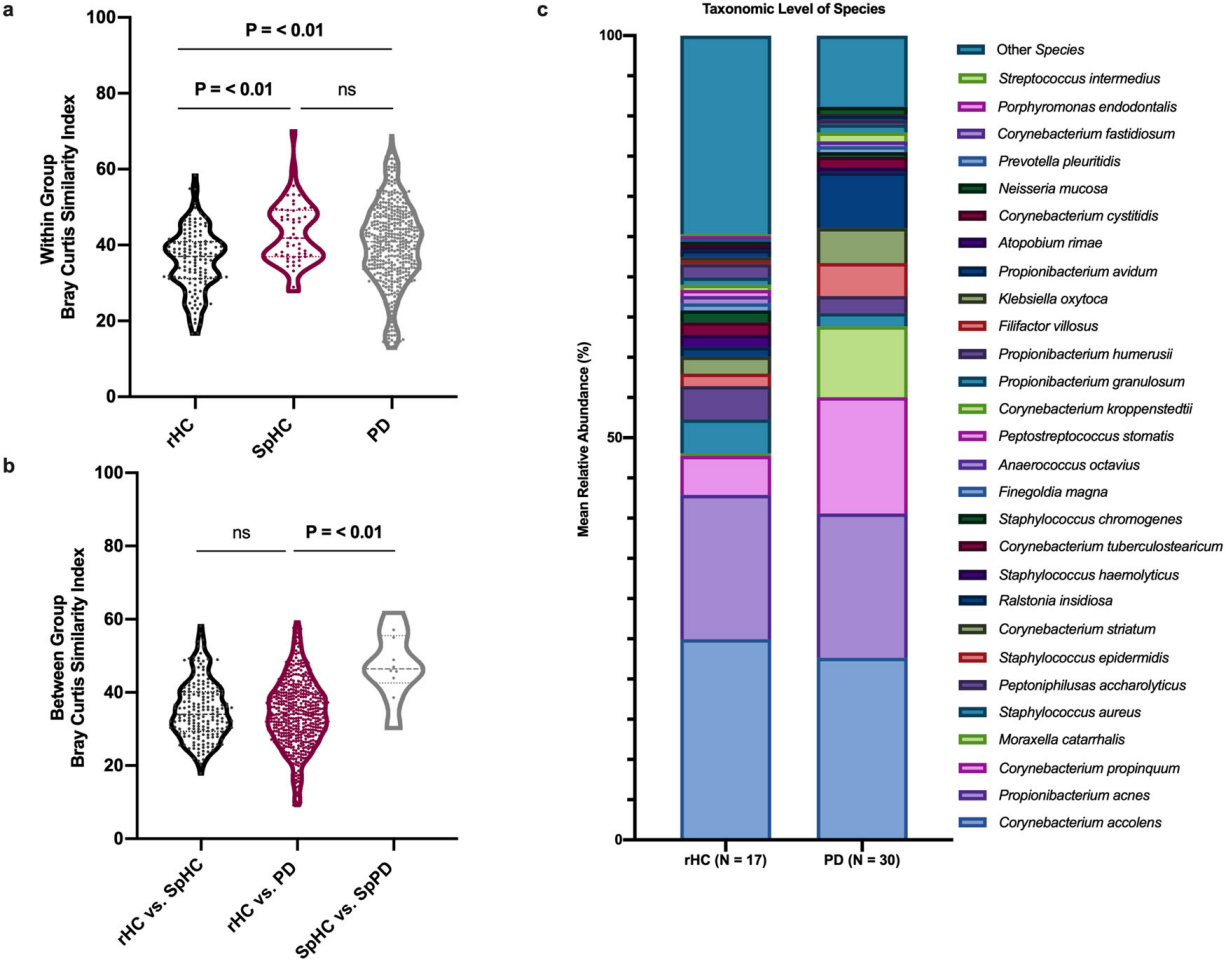
Deep nasal microbial communities in PD and control subjects. **a** Differences in overall microbial profiles (taxonomic level of species) measured using within group Bray–Curtis similarity index between random HC (rHC; *n* = 17), Spousal HC (SpHC; *n* = 11) and PD (*n* = 30; One-Way ANOVA). **b** Overall distribution of between group Bray–Curtis indices between rHC (*n* = 17) vs SpHC (*n* = 11); rHC (*n* = 17) vs. PD (*n* = 30); and SpHC (*n* = 11) vs. SpPD (*n* = 11; One-way ANOVA). Significant *p*-values (*P* < 0.05) bold. Each dot indicates a comparison of one pair of samples. **c** Average species-level relative abundance profiles of deep nasal microbiota of rHC (*n* = 17) and PD (*n* = 30). Taxa with greater than 1% relative abundance are shown.

Microbial alpha diversity was measured for each sample to determine if there were differences in microbial community structure between rHC (*n* = 17) and SpHC (*n* = 11) (Supplementary Table 1). No significant differences in alpha diversity (Shannon/Simpson index, Richness, and Evenness) were observed for analyses conducted at the taxonomic levels of phylum, genus and species. Beta-diversity analyses revealed significant differences in nasal microbial community structure between rHC and SpHC samples at the taxonomic levels of genus and species (ANOSIM: Fig. 1a, b; Supplementary Table 2). Secondary analysis of microbial community structure between rHC and SpHC samples was performed using PERMDISP and revealed a significant difference (FDR-P < 0.05) at the taxonomic level of phylum though no significant differences were observed using PERMANOVA (Supplementary Tables 3–4). Microbial taxa differing in abundance between rHC and SpHC were identified using DESeq2 and ANCOM (Supplementary Tables 5–7). A total of 41 species were significantly differentially abundant (q value < 0.01) between rHC and SpHC subjects (Supplementary Table 7; Supplementary Fig. 1). ANCOM analyses identified two differentially abundant taxa, including *Burkholderia xenovorans* (W score = 256; higher in rHC) and *Acinetobacter guillouiae* (W score = 239; higher in SpHC) (Supplementary Table 7). As the two control subject groups had significantly different deep nasal microbial communities, comparisons with PD subjects were performed separately with rHC and SpHC.

### Comparison of nasal microbiota between random non-household healthy controls (rHC) and Parkinson’s disease (PD) subjects

Microbial alpha-diversity indices were compared between rHC (*n* = 17) and PD (*n* = 30) cohorts. Indices were not significantly different between groups with the exception of species-level richness which was significantly lower in PD compared to rHC subjects, (Supplementary Table 8). Nasal microbial communities differed between rHC and PD subjects, and this manifested at taxonomic levels of genus and species, but not phylum (Fig. 1a, b; Supplementary Tables 2, 3). Taxon-by-taxon analyses also identified significantly differently abundant genera and species between groups (Fig. 1c; Supplementary Tables 9–11). Overall, 59 species were significantly differentially abundant (FDR-P < 0.01) between rHC and PD groups. The PD subjects had significantly higher abundances of putative pro-inflammatory species *Moraxella catarrhalis* and *Ralstonia insidiosa* and lower abundances of species *Blautia wexlerae, Lachnospira pectinoschiza*, and *Propionibacterium humerusii*, which are known to be capable of SCFA production in the gastrointestinal environment, and could produce SCFAs using nasal cavity mucus^12^. The PD subjects also had lower abundances of nasal mucosal anti-inflammatory *Corynebacterium* species (DESeq2; Supplementary Fig. 2; Supplementary Table 11). ANCOM identified only *Acinetobacter guillouiae* as significantly abundant in PD subjects relative to rHC subjects (W score = 290; Supplementary Table 11). A machine learning approach (Boruta) was employed for feature selection to identify taxa driving differences between PD and rHC subjects. This analysis identified eights species, including: *Escherichia albertii*, *Peptoniphilus asaccharolyticus*, *Staphylococcus aureus*, *Macrococcus brunensis*, *Ralstonia insidiosa*, *Staphylococcus epidermidis*, *Burkholderia xenovorans*, and *Acinetobacter guillouiae* (Supplementary Fig. 3).

**Fig. 2:**
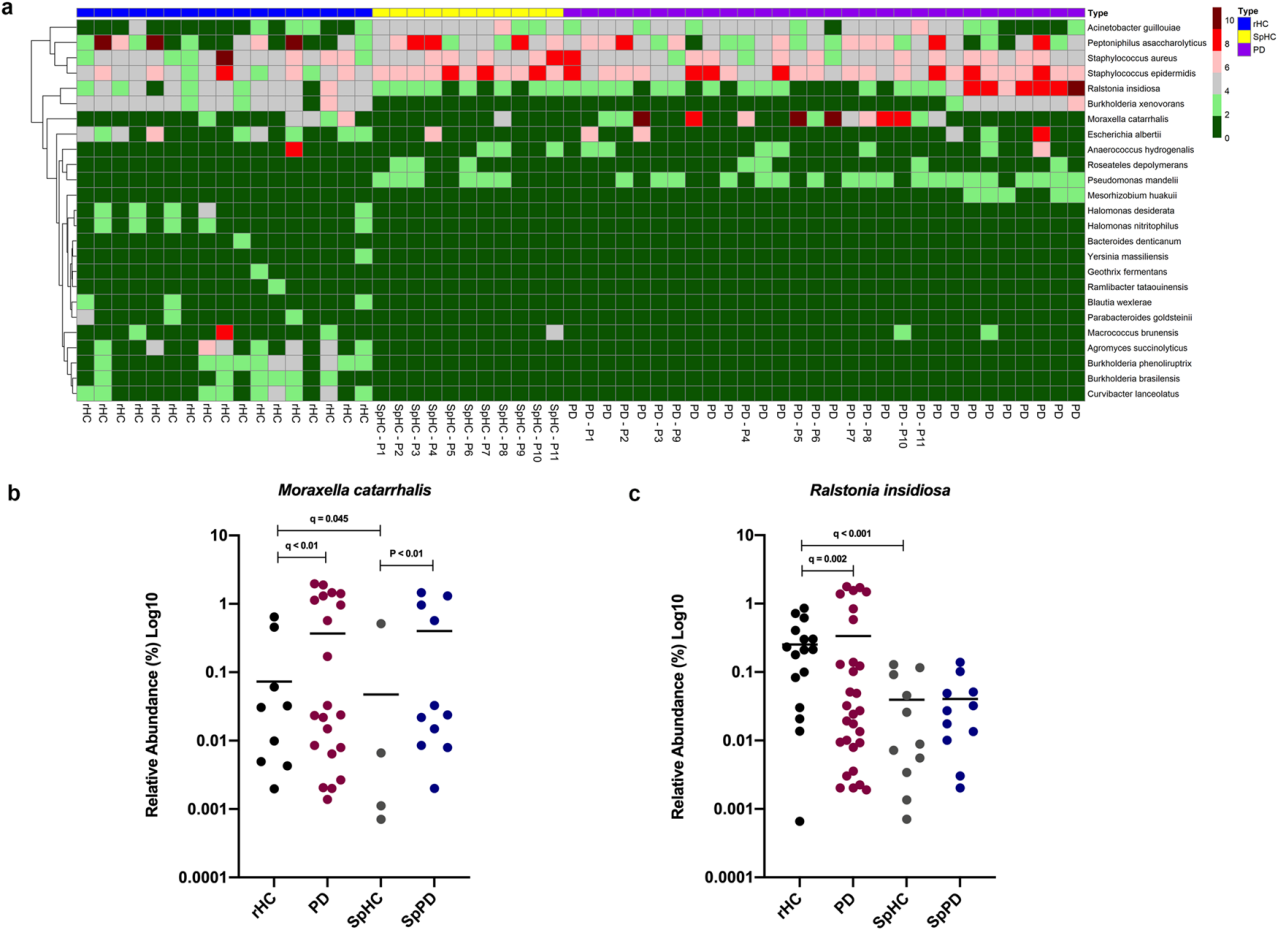
Differentially abundant deep nasal bacterial taxa between PD and control subjects. **a** Heatmap showing log10 relative abundance of deep nasal bacterial species in rHC (*n* = 17), SpHC (*n* = 11), and PD (*n* = 30) subjects. Rows include bacterial taxa that are FDR-P significant with greater than log2 fold change and identified in the Boruta feature selection algorithm for discernment of PD and rHC microbiomes. The color intensity indicates the direction of effect of relative abundance within samples. Range of colors from pink to red indicate relative abundance values (6–10) and a range from green to gray indicate relative abundance values (0–4). Each column represents a single sample, and the subject group of each sample is indicated by the top row (rHC (blue), SpHC (yellow), and PD (purple)). Each household pair (SpHC and SpPD) are labeled accordingly with its specific pair number. Relative abundance of **b** *Moraxella catarrhalis* and **c** *Ralstonia insidiosa* in rHC (*n* = 17), PD (*n* = 30), SpHC (*n* = 11), and SpPD subjects (*n* = 11) are shown. Relative abundance values are displayed as Log(x + 1) on a Log10 scale with *y*-axis starting at 0.0001, due to bimodal distribution. DESeq2 (q-values) or Wilcoxon-signed rank paired test (*p*-values) are indicated.

*M. catarrhalis* and *R. insidiosa*, identified both by DESeq2 analysis and Boruta feature selection, were elevated in PD subjects (Fig. 2a–c). However, the presence and abundance of these taxa was highly variable (bimodal distribution) within the PD cohort. The relative abundance of *M. catarrhalis* ranged from below 0.01 to a maximum of 91.46%, and for 9 of 30 PD samples, no *M. catarrhalis* sequences were detected. Many of the PD samples without *M. catarrhalis* had an elevated abundance of *Ralstonia insidiosa* (from below 0.01 to 58.21%; Fig. 2a, c).

### Comparison of deep nasal microbiota between spousal household healthy controls (SpHC) and household PD (SpPD) subjects

**Fig. 3:**
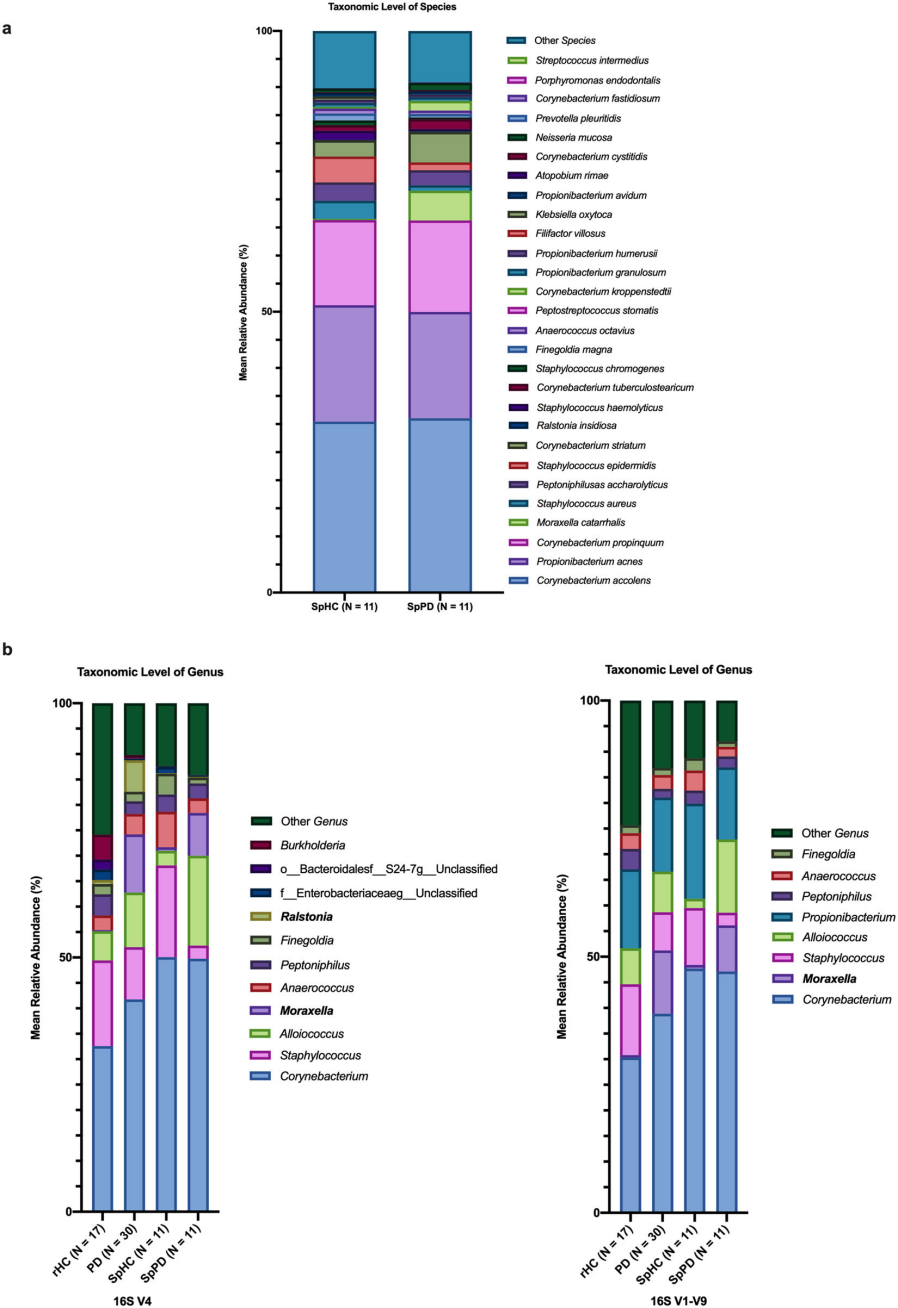
Mean relative abundances of the deep nasal microbiota between spousal PD and spousal healthy control subjects, and comparison of two different 16S rRNA gene amplicon sequencing protocols. **a** Bar plot of the deep nasal microbial communities of SpHC and SpPD subjects. The mean relative abundance of species with greater than 1% average relative abundance are shown. **b** Bar plots of the deep nasal microbial communities of rHC, PD, SpHC, and SpPD subjects using the single amplicon (V4) sequencing protocol and the multi-amplicon (Swift) sequencing protocol. Bar plots are shown for the mean deep nasal microbial community of each group at the taxonomic level of genus. Only those taxa with at least 1% average relative abundance are shown.

To determine if household spouses could serve as appropriate controls, the nasal microbiome of PD subjects’ spouses (SpHC; *n* = 11) living in the same household were compared to their paired PD subjects (SpPD; *n* = 11). The nasal microbial communities of SpHC and SpPD were more similar to each other than the general PD subjects (*n* = 30) and rHC subjects (*n* = 17), as assessed by between group Bray–Curtis similarity (Fig. 1b). No significant differences in alpha diversity between SpHC and SpPD cohorts were observed for analyses conducted at the taxonomic levels of phylum, genus, and species (Supplementary Table 12). The SpHC and SpPD nasal microbial community structures were not significantly different at the taxonomic levels of genus and species (Supplementary Tables 2–4). The nasal microbial communities of SpHC and SpPD cohorts were similar in composition, with similar average relative abundance of species *Corynebacterium accolens*, *Propionibacterium acnes*, and *Corynebacterium poropinquum* (Fig. 3a). However, the relative abundances of species *M. catarrhalis* was significantly higher, and that of *Staphylococcus epidermidis* and *Staphylococcus aureus* significantly lower, in SpPD compared to SpHC subjects (Fig. 3a). In total, 11 significantly differentially abundant species (Wilcoxon-signed rank paired test: *P* < 0.05) were detected, including a significantly higher relative abundance of *M. catarrhalis* in SpPD subjects (Fig. 2b; Supplementary Table 13). Boruta feature selection identified five bacterial species that were driving differential SpPD microbiomes from SpHC microbiomes. These species included: *Staphylococcus aureus*, *Staphylococcus chromogenes*, *Trabulsiella farmeri, M. catarrhalis*, and *Staphylococcus epidermidis* (Supplementary Fig. 4).

### Correlation of nasal microbiota with clinical characteristics in PD subjects

We investigated whether clinical characteristics like age, PD duration, motor symptom severity (MDS-UPDRS, H&Y), olfactory function (UPSIT) and levodopa daily dosage (LEDD) could be significantly correlated with bacterial taxa. Univariate analysis, using Pearson’s correlation, was conducted on 29 PD subjects with clinical metadata and microbial features at the taxonomic levels of phylum and species.

**Fig. 4:**
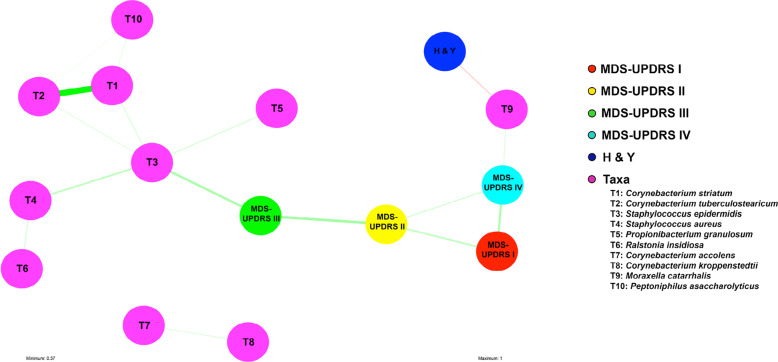
Multivariate analysis of bacterial species and PD clinical features. Visualization of multiple associations present in clinical features of PD subjects with deep nasal microbiota data using a multivariate network analysis of bacteria (species) in the deep nasal sinus of PD subjects (*n* = 29) and PD clinical characteristics. Positive correlations (green arrows), negative correlations (red arrows), strong (thick edges) and weak (thin edges, less saturated) correlations between PD subject clinical features and bacterial species are shown. Correlation arrows displayed are significant (*P* < 0.05) and have R-values greater than 0.46.

MDS-UPDRS IV scores positively correlated with Proteobacteria (R = 0.39, R^2^ = 0.15, *P* = 0.04), while H&Y scores were negatively correlated with Proteobacteria (R = −0.50, R^2^ = 0.26, *P* = 0.006). Three species were significantly correlated with PD subject clinical characteristics (Table 1, Supplementary Fig. 5). H&Y scores were negatively correlated with *M. catarrhalis*. MDS-UPDRS subject scores positively correlated with *M. catarrhalis* and *Staphylococcus epidermidis*. UPSIT scores positively correlated with the commensal bacterial species *Peptinophilus asaccharolyticus*. A total of 21 out of 30 PD subjects were taking LEDD. Our results indicated no significant (*P* > 0.05) correlations between the PD subjects’ LEDD and the most abundant phyla or species (>1%). A network analysis of the most abundant microbial features (>1% relative abundance across all samples) was used to visualize interactions between measured PD clinical features (e.g., UPDRS) and the nasal microbiome (Fig. 4; Supplementary Fig. 6). This analysis identified correlational clusters between *Ralstonia insidiosa*, *Staphylococcus aureus*, and *Staphylococcus epidermidis*, and between *Corynebacterium striatum*, *Corynebacterium tuberculostearicum*, and *Peptoniphilus asaccharolyticus* (Fig. 4).

**Table 1:** Altered deep nasal microbial community features correlated with clinical variables of PD subjects.

	Taxa	Taxonomic level	R value	R-squared value	*p*-value
H & Y	*Moraxella catarrhalis*	Species	−0.46	0.21	**0.01**
MDS-UPDRS III	*Staphylococcus epidermidis*	Species	0.57	0.33	**<0.01**
MDS-UPDRS IV	*Moraxella catarrhalis*	Species	0.44	0.19	**0.02**
UPSIT	*Peptoniphilus asaccharolyticus*	Species	0.48	0.23	**0.04**

Clinical characteristics of study PD subjects, including H & Y, MDS-UPDRS III, IV, and UPSIT, were correlated with the species taxonomic level of PD subjects' deep nasal microbiome (*N* = 29) using Pearson’s correlation on rarefied data. R = Pearson’s correlation coefficient, ranging from values +1 to −1. R-squared value = the square of Pearson’s correlation coefficient. Significant *P*-values (*P* < 0.05; R > ±0.3: bold).

### 16S rRNA V4 sequencing analysis

To validate our findings obtained using the multi-amplicon sequencing approach as shown above, we performed a more conventional 16S rRNA amplicon sequencing analysis targeting only a single variable region (V4). Using the same analytical approach, all group comparisons were performed again with the V4 dataset, at the taxonomic levels of family and genus due to lower taxonomic resolution of the single amplicon data (Supplementary Fig. 7; Supplementary Tables 14–21). At the taxonomic level of genus, both methods yielded highly similar observed microbial communities, with the exception that the V4 amplicon did not properly amplify bacteria from the genus *Propionibacterium* (Fig. 3b, Supplementary Tables 22–24). This phenomenon has been identified previously and is due to mismatches between the rRNA gene of bacteria from the genus *Propionibacterium* and the commonly used 515 F and 806 R primers at or near the 3′ ends of each primer^13^.

Analysis of the single amplicon sequence data also indicated that PD subjects had higher relative abundance of bacteria from the genus *Moraxella* compared to rHC (q < 0.01; Supplementary Table 23). Additionally, the relative abundance of *Staphylococcus* was lower in SpPD relative to SpHC subjects (Wilcoxon-signed rank paired test: *P* < 0.05) (Supplementary Table 24).

## Discussion

This proof-of-concept study shows that PD subjects have a dysbiotic microbial community in the deep nasal sinus cavity characterized by loss of putative beneficial bacteria and an increase in putative pro-inflammatory bacteria. Since nasal middle meatus mucosa are in close proximity to the olfactory bulb, our findings of a putative pro-inflammatory mucosal-associated microbiota community in the deep nasal sinus cavity supports Braak’s hypothesis that the olfactory bulb is one of the sites that initiates and/or perpetuates neuroinflammation and alpha-synuclein aggregation in PD^5^. Our data warrant future investigation into the exact role of deep nasal sinus cavity microbiota inflammation in PD pathogenesis.

To date, no study has compared the deep nasal sinus cavity microbial profiles between PD and control subjects. The deep nasal microbiota community in PD subjects was distinct when compared to either spousal or random healthy controls, with changes characterized by increased relative abundances of putative pro-inflammatory bacteria belonging to phylum Proteobacteria, including *M. catarrhalis*. The increased relative abundance of this taxon in PD subjects was confirmed by two distinct 16S rRNA gene amplification protocols. *M. catarrhalis* is an LPS-producing bacterium (phylum Proteobacteria), and while members of the genus were previously regarded as human commensals^14^, they are now emerging as pathogens causing infections in the eyes, ears, upper respiratory tract, and joints^15^. Although the presence of *Moraxella* was not always associated with the nasal microbiome of PD subjects, an increased relative abundance of *M. catarrhalis* in PD subjects (median = 0.019%; range of <0.01–91.46%), as well as other pathogens, suggests a role promoting nasal inflammation and possibly neuroinflammation in PD. The microbiomes with the highest relative abundance of *Moraxella* (e.g., greater than 10%) were consistently from PD subjects.

Another feature of the deep nasal sinus cavity microbiota community in PD subjects was reduced relative abundance of putative anti-inflammatory bacteria, including *Blautia wexlerae, Lachnospira pectinoschiza*, and *Propionibacterium humerusii*. These three taxa have been characterized as anti-inflammatory SCFA-producing bacteria associated with reduced abundance in PD gut microbiome and other inflammatory disorders such as colorectal cancer^16^. Thus, these organisms and other similar taxa may play an important role in maintaining a balanced (anti-inflammatory) microbial composition in the nasal microbiome. This model of loss of beneficial commensals resulting in overgrowth of pathobionts is common to many human diseases associated with dysbiotic microbiomes^17^.

The putatively dysbiotic microbial profiles in PD subjects observed in this study have broad similarities to profiles observed in studies of gut microbiota in PD, as summarized in detail in recent reviews^18,19^. Collectively, those studies have revealed increased pro-inflammatory/LPS-producing bacteria and a reduction in putative beneficial/SCFA-producing bacteria in PD^3,18^. Thus, our study supports the hypothesis that gut and deep nasal dysbiotic microbiota communities are triggers/enablers of neuroinflammation, and this could lead to alpha-synuclein aggregation and dopamine loss in the brain.

In contrast to our findings, two recent studies using superficial nostril swabs or nasal fluid wash demonstrated no clear differences within the nasal microbiome of PD subjects vs. random HC subjects^10,11^. We posit that collecting nasal swabs from the deep nasal cavity, under endoscopic guidance, results in sampling of a unique and more biologically relevant site. This specific collection method allows for the nasal mucosal microbial composition in close proximity to the olfactory bulb to be examined. This location is more likely to impact PD pathology, consistent with Braak’s hypotheses and PD subject autopsy data^7^. Prior studies have demonstrated that microbial communities are distinct in different parts of the body including different skin sites^20^, multiple locations along the GI tract^21^, and different locations within the oral cavity^22^. Similarly, we hypothesized that the deep nasal sinus cavity microbiome is unique, and that data from the oro-nasal cavity may not sure as a viable proxy for the deep nasal sinus cavity microbiome. Further studies will be required to better understand the role of deep nasal sinus cavity microbiome.

Our results demonstrate that specific bacterial species may correlate with certain PD phenotypes. For instance, we found that *M*. *catarrhalis* positively correlated with MDS-UPDRS IV scores, but negatively correlated with H&Y scores. The MDS-UPDRS Part IV measures motor complications of PD, while H&Y staging is weighted heavily towards postural instability^23,24^. Thus, the positive correlation of *M. catarrhalis* with MDS-UPDRS Parts IV and negative correlation with H&Y staging suggests a phenotype where subjects exhibit more prominent motor symptoms, but with potentially preserved postural stability. Similar microbial and phenotypic relationships were found with the phyla Proteobacteria. Additional studies are needed to verify these correlations between species and PD phenotype. Moreover, *M. catarrhalis* co-occurred with clusters of opportunistic pathogens species including *Ralstonia insidiosa*, *Staphylococcus aureus*, and *Staphylococcus epidermidis*; these taxa also positively correlated with PD motor scores. Unsurprisingly, olfactory function was negatively correlated with disease duration. Although olfactory function did not correlate with the relative abundance of any putative pro-inflammatory bacteria, olfactory function was positively correlated with the commensal *Peptinophilus asaccharolyticus*. Within the multivariate analysis, *Peptinophilus asaccharolyticus* clustered with species *Corynebacterium striatum*, and *Corynebacterium tuberculostearicum*. The role of these organisms in the host function of smell is not known. Bacteria from the genus *Corynebacterium* are dominant in the sinonasal microbiome of healthy individuals^25^. However, their abundance is negatively affected by chronic inflammation in the upper airways, and they exhibit an inverse relationship with *Staphylococcus aureus*^26^. Therefore, the decrease of *Corynebacterium* spp. in PD subjects with smell loss suggests an important role in maintaining nasal mucosal homeostasis and preventing inflammation. These data support microbial species as a prediction model for PD using a feature selection algorithm and demonstrate a correlation of specific nasal microbiota with worsened PD clinical characteristics. Further studies are also needed to confirm our observation of the significant positive correlation between *M. catarrhalis* and PD disease severity.

We showed that microbial communities in PD are more similar to their household spousal healthy controls (SpHC) than age/gender/race matched random healthy controls. The similarity of the nasal microbiome in SpPD and SpHC subjects is consistent with prior studies showing that individuals who share a common household share similar microbiomes^27,28^. Additionally, several studies have demonstrated that microbial diversity changes in the nasal microbiome are dependent upon environmental conditions such as living quarters and pets^27^. Our data provided evidence that at least some of the alterations in the microbiota community (including gut microbiota community) reported in PD subjects might be a consequence of unique environmental factors associated with the PD lifestyle. Our data supports the use of household controls in future studies of the microbiota community. We note that despite the increased similarity of SpPD and SpHC microbiomes, bacteria from the genus *Moraxella* were still significantly different between groups, and this suggests a physiological rather than environmental driver.

This study’s limitations include the relatively small sample size and lack of inclusion of ambient air samples and surface samples of the households of spousal participants. The difficult sample collection, performed by RUMC rhinologists experienced in nasal sample collection after complete nasal endoscopy and using endoscopy guided small sized nasal swabs from the middle meatus, restricted recruitment of large numbers of subjects in the PD, SpHC, and rHC groups. Abnormal microbiomes in the deep nasal sinus cavity have been reported in patients with anosmia^29^. Koskinen et al. posit that the nasal microbiome could shape olfactory function and thus, dysbiotic microbiota in patients with abnormal smell could be a contributing factor in olfactory bulb dysfunction. This is the only published study that has interrogated microbiota community in the deep nasal sinus cavity—a testament to the technical difficulty in obtaining samples from this site. However, we note that the sample size (*n* = 67) employed by Koskinen et al. is similar to that of our study (*n* = 58).

In conclusion, our proof-of-concept study provides preliminary data indicating the presence of a dysbiotic and potentially pro-inflammatory deep nasal sinus cavity microbiota environment in PD subjects as compared to rHC and SpHC subjects. Within PD subjects there was a positive correlation between putative pro-inflammatory bacteria, including *M. catarrhalis*, and PD clinical features. Furthermore, the value of including household controls for studying microbial community structure in disease states such as PD is confirmed. Overall, our study supports a strong scientific rationale for future mechanistic studies to establish a causal link between nasal microbiota dysbiosis and PD pathogenesis

## Methods

### Study design

This cross-sectional case-control study was conducted at Rush University Medical Center (RUMC) Chicago, IL. We aimed to interrogate the microbial community of the middle meatus nasal cavity of PD subjects and compare them to (1) their healthy spousal household control counterparts, and (2) random, age/gender/race matched healthy controls. All subjects signed the RUMC Institutional Review Board approved informed consent form, and the study was registered with National Institute of Health (NIH) Clinical Trials (ClinicalTrials.gov Identifier: NCT03336697).

### Subject’s demographics and clinical characteristics

PD subjects were recruited from the RUMC Movement Disorders Clinic. PD subjects met the United Kingdom Parkinson’s Disease Society Brain Bank diagnostic criteria^30^ and were required to be Hoehn & Yahr (H&Y) stage 1–3 at the time of enrollment. Spousal healthy controls of PD subjects (SpHC) were also invited to participate. Random non-spousal healthy control subjects (rHC) were recruited via research advertisements at RUMC. All healthy control subjects were matched to PD subjects according to age (±5 years) and sex. All control patients were healthy non-allergic patients who were evaluated by otolaryngologist and had no evidence of chronic inflammation that might impact the nasal microbiota.

Subjects were excluded based on the following criteria: (1) Occupation expected to change intestinal flora pattern (e.g., sanitation worker); (2) Treatment with medications that may induce parkinsonism including metoclopramide; (3) Typical or atypical antipsychotic medications; (4) Treatment within 12 weeks with systemic and nasal antibiotics, probiotics and prebiotics; (5) Known diagnosis of inflammatory bowel disease; symptomatic organic gastrointestinal (GI) disease other than hemorrhoids and hiatal hernia; (6) Abdominal surgeries for GI disease such as bowel resection, diverticular surgery, colostomy (surgery for hemorrhoids and cholecystectomy or appendectomy for benign disease more than 5 years prior to enrollment were allowed); (7) Symptomatic functional GI disease that could impair intestinal motility such as scleroderma or use of GI motility drugs; (8) Acute illness requiring hospitalization; (9) Pre-existing organ failure or comorbidities as these may change GI flora, like liver disease, chronic kidney disease, uncontrolled psychiatric illness; (10) Clinically important lung disease or heart failure; (11) HIV disease; (12) Alcoholism, unreliable drinking history or consumption of alcohol more than three times a week or binge drinking or drinking more than or equal to three drinks per occasion; (13) Transplant recipients; (14) History of diabetes; (15) Clinically significant dehydration or clinically detectable ascites or peripheral edema or cardiac failure; (16) Presence of short bowel syndrome or severe malnutrition; (17) Use of immunosuppressive medications with three months of enrollment; (18) Chronic use of diuretics.

A movement disorders neurologist examined all PD and SpHC subjects. Parkinsonian symptoms were assessed using the Movement Disorders Society Revision of the Unified Parkinson’s Disease Rating Scale (MDS-UPDRS) I–IV^23,24^ and H&Y staging^23^. Olfactory function was measured using University of Pennsylvania Smell Identification Test (UPSIT)^31^.

Fifty-eight (*n* = 58) participants were enrolled, including 30 PD subjects and 28 healthy control subjects. Of the 28 healthy control subjects, 11 were SpHC of 11 corresponding PD subjects (SpPD), while the remaining were 17 rHC (Table 2). There were no significant differences in age or sex between any groups. However, significantly higher numbers of men were recorded in the rHC group then in the SpHC group (71% vs. 45%, *p* ≤ 0.01). Additionally, we report mean and standard deviations for the following clinical features: age of onset of disease, disease duration, MDS-UPDRS I–IV, H&Y Stages, levodopa daily dosages (LEDD), and UPSIT (Table 2). The metadata for all individual subjects reported in this study are shown in Additional File 1.

**Table 2:** Clinical features of Parkinson’s disease subjects and healthy control subjects.

	PD	rHC	*p*-value	SpPD	SpHC	*p*-value
Total subjects	30	17	–	11	11	–
Age	60.12 (6.86)	55.18 (11.09)	0.64	58.4 (5.09)	59.4 (5.2)	0.65
Sex (% male)	83%	71%	0.46	64%	45%	0.67
Age of onset	54.69 (8.15)	–	–	53.2 (7.88)	–	–
Disease duration	5.53 (4.81)	–	–	14.1 (19.78)	–	–
Total MDS-UPDRS	30.1 (14.1)	–	–	26.67 (9.32)	–	–
MDS-UPDRS I	5.5 (3.2)	–	–	5.58 (3.26)	–	–
MDS-UPDRS II	5.4 (4.9)	–	–	5 (2.86)	–	–
MDS-UPDRS III	17.8 (8.3)	–	–	14.25 (4.47)	–	–
MDS-UPDRS IV	1.3 (2.5)	–	–	1.83 (2.79)	–	–
H&Y Stages	1.9 (0.3)	–	–	1.82 (0.40)	–	–
LEDD	400.8 (369.6)	–	–	308.18 (245.72)	–	–
UPSIT	24.7 (6.7)	–	–	26.2 (8.36)	37 (2.7)	**0.02**

58 participants; Mean ± (S.D.). Statistical analyses were performed using the Students *t*-test (age), Chi-square test (sex), and Mann–Whitney U (UPSIT). Significant *P*-values (*P* < 0.05: bold).

*MDS-UPDRS* Movement Disorder Society’s Unified Parkinson’s Disease Rating Scale, *H&Y* Hoehn and Yahr, *LEDD* Levodopa Equivalent Daily Dosing, *UPSIT* The Smell Identification Test, *PD* Parkinson’s disease, *rHC* random non-spousal healthy controls, *SpPD* household Parkinson’s disease, *SpHC* household spousal healthy controls.

### Sample collection, DNA extraction, and DNA sequencing

We used deep nasal swabbing under nasal anterior endoscopy by a trained rhinologist in the Department of Otorhinolaryngology at RUMC. Utilizing a 30-degree rigid nasal endoscope, the physician gently passed a cotton swab into the nasal passage to visualize and swab the olfactory cleft region. The cotton swab heads were placed in sterile tubes and frozen immediately at −80 °C until DNA extraction. Total genomic DNA was extracted from the swabs using the FastDNA SPIN Kit from the manufacturer’s protocol (FastDNA Spin Kit for Soil, MP Biomedicals, Solon, OH). DNA concentrations were measured with fluorometric quantitation (Qubit, Life Technologies, Grand Island, NY, USA) (Supplementary Table 25), PCR amplified and sequenced on an Illumina MiniSeq sequencer at the Genome Research Core (GRC) at the University of Illinois at Chicago (UIC). 16S rRNA gene amplicon library preparation was performed using two parallel techniques. To provide superior resolution at the species taxonomic level, a multi-amplicon workflow was conducted employing the Swift Amplicon 16S + ITS Panel (Swift Biosciences, Madison, WI). The primary microbiome analyses were performed on this dataset and results are based on these data unless otherwise stated. Libraries were also prepared using a standard single target 16S rRNA gene amplicon pipeline, targeting the V4 region of the microbial small subunit rRNA gene with primers 515 F/806R^32^, as described previously^33^. To reduce batch effects, all samples were extracted using the same DNA extraction kit at the same time, and library preparation for all samples was conducted in 96-well plates simultaneously. Raw sequence data (FASTQ files) were deposited in the National Center for Biotechnology Information (NCBI) Sequence Read Archive (SRA), under the BioProject identifier PRJNA625973 (Swift multi-amplicon) and PRJNA625976 (V4 amplicon).

### 16S rRNA V1–V9 sequencing analysis

Annotation of the multi-amplicon data was performed using the 16S Metagenomics application (v1.0.1) within the Illumina Basespace cloud computing environment and employing the Illumina-curated version of the GreenGenes reference database. The primary output from the dataset was a biological observation matrix (BIOM)^34^ generated at each taxonomic level from phylum to species. Downstream analyses were performed using the software package Primer7^35^ and packages within the R programming environment (R Core Team, 2017).

### 16S rRNA V4 sequencing analysis

Raw sequences obtained were merged using the PEAR (Paired-End read merger) algorithm (v0.9.11)^36^. Merged sequences were then quality filtered using cutadapt and denoised using the DADA2 algorithm within the QIIME2 (v 2020.8.0) workflow^37–40^. The produced amplicon sequence variants (ASVs) were used in all downstream analyses. Taxonomy was assigned to ASVs by using the naive Bayes taxonomy classifier against the SILVA_138 99% out database reference sequences^41–43^. No reagent contaminant ASVs were identified using decontam package based on prevalence in the PCR negative control samples, using default parameters^44^. Chloroplast, aquatic, soil, and mitochondrial ASVs were removed from statistical analysis^45^.

### Statistical analysis

Analyses of alpha- and beta-diversity were used to examine changes in nasal microbial community structure. Alpha-diversity metrics (i.e., Shannon index, Simpson’s index, richness and evenness) were calculated from rarefied datasets (5000 sequences/sample for V1–V9 amplicons; 2300 sequences/sample for V4 amplicons). Three sets of comparisons were made: (1) PD subjects and non-spousal (random) controls, (2) PD subjects and their spousal control counterparts, and (3) spousal controls and non-spousal controls. Group comparisons were performed with Students *t*-test, Chi-square test, and Mann–Whitney U test for quantitative and categorical variables as appropriate using the software GraphPad Prism (v9.0, GraphPad Software LLC San Diego California).

Pairwise Bray–Curtis dissimilarity was used as the metric for data visualization and analysis of similarity (ANOSIM) calculations to assess microbial community structure differences between samples. ANOSIM was performed at the phylum, genus and species taxonomic levels on square-root transformed data. In addition, Permutation Multivariate Analysis of Variance (PERMANOVA) (ideal for equal sample size group comparisons) and Permutational Analysis of Multivariate Dispersions (PERMDISP) (ideal for unequal sample size group comparisons) were utilized to assess the microbial composition between groups at the phylum, genus, and species taxonomic levels^46–48^. Significance of PERMANOVA and PERMDISP values were determined using 9999 permutations and corrected for multiple testing using the Benjamini–Hochberg method (q < 0.05).

Differential abundances of individual taxa between groups were determined using differential abundance analysis (DESeq2)^49,50^ generating a false-discovery rate (FDR) corrected p-value. DESeq2 has been shown to most appropriate for differential abundance comparisons in studies with small sample size groups (<20) or unbalanced design^50^. DeSeq2 multi-amplicon data was filtered to include individual taxa with an absolute log2 fold change (>±1.2) with a significant FDR-P value (q < 0.01). Additionally, analysis of composition of microbiomes (ANCOM) was performed on the nasal microbial community between subject groups to identify differentially abundant taxa in analyses of compositional data^51,52^. This analysis generates a W score, which counts of the number of sub-hypotheses that have passed for a given taxon. ANCOM samples with less than 5000 (V1–V9) or 2300 (V4) sequences were removed. All listed significant features [genus and species] rejected the null hypothesis. Furthermore, the Wilcoxon-signed rank test, using R programming, was utilized to determine the differences in the relative abundance of shared taxa for paired analyses between spouses.

A network analysis was generated using the *qgraph* package within the R programming language. Pearson’s correlations were generated between the relative abundances of specific taxa (phylum and species) with PD clinical parameters with a significant threshold of *p*-value: (*p* < 0.05) and R value: (>0.3). Random forest models (number of runs = 1000) were used to predict disease status based on the microbiota profile using the R implementation of the algorithm (Boruta algorithm, “randomForest” package)^53^.

### Reporting summary

Further information on research design is available in the Nature Research Reporting Summary linked to this article.

## Supplementary information

Supplementary Information

Supplementary Data 1

Reporting Summary

## Data Availability

Raw sequence data (FASTQ files) were deposited in the National Center for Biotechnology Information (NCBI) Sequence Read Archive (SRA), under the BioProject identifier PRJNA625973 (Swift multi-amplicon: 16S V1–V9) and PRJNA625976 (16S V4 amplicon). All other relevant data are available from the corresponding author upon reasonable request.

## Code availability

The code to generate microbial analysis for Swift multi-amplicon (16S rRNA V1–V9) and 16S rRNA V4 amplicon sequencing has been deposited at the Zenodo repository, with the following https://zenodo.org/record/5039413#.YNo_XC1h1pR.

## References

[1] Kalia LV, Lang AE (2015). Parkinson’s disease. Lancet.

[2] Hawkes CH, Del Tredici K, Braak H (2007). Parkinson’s disease: a dual-hit hypothesis. Neuropathol. Appl. Neurobiol..

[3] Unger MM (2016). Short chain fatty acids and gut microbiota differ between patients with Parkinson’s disease and age-matched controls. Parkinsonism Relat. Disord..

[4] Braak H, Rüb U, Gai WP, Del Tredici K (2003). Idiopathic Parkinson’s disease: possible routes by which vulnerable neuronal types may be subject to neuroinvasion by an unknown pathogen. J. Neural Transm..

[5] Braak Heiko, De Vos RAI, Bohl J, Del Tredici K (2006). Gastric α-synuclein immunoreactive inclusions in Meissner’s and Auerbach’s plexuses in cases staged for Parkinson’s disease-related brain pathology. Neurosci. Lett..

[6] Haehner A, Masala C, Walter S, Reichmann H, Hummel T (2019). Incidence of Parkinson’s disease in a large patient cohort with idiopathic smell and taste loss. J. Neurol..

[7] Rawls M, Ellis AK (2019). The microbiome of the nose. Ann. Allergy Asthma Immunol..

[8] Leboucq N, Menjot De Champfleur N, Menjot De Champfleur S, Bonafé A (2013). The olfactory system. Diagn. Intervent. Imaging.

[9] Yang HJ (2018). Association of nasal microbiome and asthma control in patients with chronic rhinosinusitis. Clin. Exp. Allergy.

[10] Heintz-Buschart A (2018). The nasal and gut microbiome in Parkinson’s disease and idiopathic rapid eye movement sleep behavior disorder. Mov. Disord..

[11] Pereira PAB (2017). Parkinsonism and related disorders oral and nasal microbiota in Parkinson’s disease. Parkinsonism Relat. Disord..

[12] Cho DY (2020). Contribution of short chain fatty acids to the growth of *Pseudomonas aeruginosa* in rhinosinusitis. Front. Cell Infect. Microbiol..

[13] Gohl DM (2016). Systematic improvement of amplicon marker gene methods for increased accuracy in microbiome studies. Nat. Biotechnol..

[14] van den Munckhof E. H. A. et al. Nasal microbiota dominated by Moraxella spp. is associated with respiratory health in the elderly population: a case control study. Respir. Res. **21**, 10.1186/s12931-020-01443-8 (2020). 10.1186/s12931-020-01443-8 PMC7362441 32664929

[15] Verduin CM, Hol C, Fleer A, Van Dijk H, Van Belkum A (2002). Moraxella catarrhalis: from emerging to established pathogen. Clin. Microbiol. Rev..

[16] Chen W, Liu F, Ling Z, Tong X, Xiang C (2012). Human intestinal lumen and mucosa-associated microbiota in patients with colorectal cancer. PLoS ONE.

[17] Kamada N, Seo S-U, Chen GY, Núñez G (2013). Role of the gut microbiota in immunity and inflammatory disease. Nat. Rev. Immunol..

[18] Lubomski M (2020). Parkinson’s disease and the gastrointestinal microbiome. J. Neurol..

[19] Keshavarzian A, Engen P, Bonvegna S, Cilia R (2020). The gut microbiome in Parkinson’s disease: a culprit or a bystander?. Prog. Brain Res..

[20] Huttenhower C, Gevers D, Knight R (2012). “Structure, function and diversity of the healthy human microbiome.”. Nature.

[21] Gilbert JA (2018). “Current understanding of the human microbiome.”. Nat. Med.

[22] Narengaowa W, Kong F, Lan UF, Awan H, Qing, Ni J (2021). “The oral-gut-brain AXIS: the influence of microbes in Alzheimer’s disease.”. Front. Cell Neurosci..

[23] Goetz CG (2004). Movement Disorder Society Task Force report on the Hoehn and Yahr staging scale: status and recommendations. Mov. Disord..

[24] Goetz CG (2008). Movement Disorder Society UPDRS Revision Task Force. Movement Disorder Society-sponsored revision of the Unified Parkinson’s Disease Rating Scale (MDS-UPDRS): scale presentation and clinimetric testing results. Mov. Disord..

[25] Hang J (2017). “Composition and variation of respiratory microbiota in healthy military personnel.”. PLoS ONE.

[26] Mahdavinia M (2018). “The nasal microbiome in patients with chronic rhinosinusitis: analyzing the effects of atopy and bacterial functional pathways in 111 patients.”. J. Allergy Clin. Immunol..

[27] Dill-McFarland KA (2019). Close social relationships correlate with human gut microbiota composition. Sci. Rep..

[28] Song SJ (2013). Cohabiting family members share microbiota with one another and with their dogs. ELife.

[29] Koskinen K (2018). “The nasal microbiome mirrors and potentially shapes olfactory function.”. Sci. Rep..

[30] Hughes AJ, Daniel SE, Kilford L, Lees AJ (1992). Accuracy of clinical diagnosis of idiopathic Parkinson’s disease: a clinico-pathological study of 100 cases. J. Neurol. Neurosurg. Psychiatry.

[31] Doty RL, Shaman P, Kimmelman CP, Dann MS (1984). University of pennsylvania smell identification test: a rapid quantitative olfactory function test for the clinic. Laryngoscope.

[32] Parada AE, Needham DM, Fuhrman JA (2016). Every base matters: assessing small subunit rRNA primers for marine microbiomes with mock communities, time series and global field samples. Environ. Microbiol..

[33] Naqib, A. et al. In *Gene Expression Analysis,* 149–169 (Humana Press, 2018).

[34] McDonald D (2012). The Biological Observation Matrix (BIOM) format or: how I learned to stop worrying and love the ome-ome. GigaScience.

[35] CLARKE KR (1993). Non-parametric multivariate analyses of changes in community structure. Aust. J. Ecol..

[36] Zhang J, Kobert K, Flouri T, Stamatakis A (2014). PEAR: a fast and accurate Illumina Paired-End reAd mergeR. Bioinformatics.

[37] Martin M (2011). Cutadapt removes adapter sequences from high-throughput sequencing reads. EMBnet. J..

[38] Callahan BJ (2016). DADA2: high-resolution sample inference from Illumina amplicon data. Nat. Methods.

[39] Bolyen E (2019). Reproducible, interactive, scalable and extensible microbiome data science using QIIME 2. Nat. Biotechnol..

[40] Estaki M (2020). QIIME 2 enables comprehensive end-to-end analysis of diverse microbiome data and comparative studies with publicly available data. Curr. Protoc. Bioinformatics.

[41] Bokulich NA, Kaehler BD, Rideout JR (2018). Optimizing taxonomic classification of marker-gene amplicon sequences with QIIME 2’s q2-feature-classifier plugin. Microbiome.

[42] Yarza P (2014). Uniting the classification of cultured and uncultured bacteria and archaea using 16S rRNA gene sequences. Nat. Rev. Microbiol..

[43] Quast C (2013). The SILVA ribosomal RNA gene database project: Improved data processing and web-based tools. Nucleic Acids Res..

[44] Davis NM, Proctor DM, Holmes SP, Relman DA, Callahan BJ (2018). Simple statistical identification and removal of contaminant sequences in marker-gene and metagenomics data. Microbiome.

[45] Hanshew AS, Mason CJ, Raffa KF, Currie CR (2013). Minimization of chloroplast contamination in 16S rRNA gene pyrosequencing of insect herbivore bacterial communities. J. Microbiol. Methods.

[46] Oksanen J (2019). Package “vegan” Title Community Ecology Package. Community Ecol. Package.

[47] Anderson MJ (2006). Distance-based tests for homogeneity of multivariate dispersions. Biometrics.

[48] Kelly BJ (2015). Power and sample-size estimation for microbiome studies using pairwise distances and PERMANOVA. Bioinformatics.

[49] Love MI, Huber W, Anders S (2014). Moderated estimation of fold change and dispersion for RNA-seq data with DESeq2. Genome Biol..

[50] Li Y, Andrade J (2017). DEApp: An interactive web interface for differential expression analysis of next generation sequence data. Source Code Biol. Med..

[51] Weiss S (2017). Normalization and microbial differential abundance strategies depend upon data characteristics. Microbiome.

[52] Mandal S (2015). Analysis of composition of microbiomes: a novel method for studying microbial composition. Microb. Ecol. Health Dis..

[53] Kursa MB, Rudnicki WR (2010). Feature selection with the boruta package. J. Stat. Softw..

