# Supplementary material for: Deep nasal sinus cavity microbiota dysbiosis in Parkinson’s disease
Source: NPJ Parkinsons Dis. 2021 Dec 8;7:111. doi: 10.1038/s41531-021-00254-y (PMC8655044; doi:10.1038/s41531-021-00254-y)
Supplement: Supplementary file 1 — Supplementary Information [file 41531_2021_254_MOESM1_ESM.pdf]

Supplementary Material

Swift 16S rRNA (V1-V9) Sequencing Analysis

Supplementary Table 1. Alpha diversity values of random non-household (rHC) and spousal household (SpHC) healthy controls subjects.

Comparison Groups	Taxonomic Levels	Diversity Index	rHC: Mean (SD)	SpHC: Mean (SD)	p value	q value
rHC (n=12) vs. SpHC (n=11)	Phylum	Shannon	1.18 (0.51)	1.11 (0.45)	0.46	0.46
rHC (n=11) vs. SpHC (n=11)	Genus	Shannon	2.48 (1.21)	2.22 (0.89)	0.72	0.72
rHC (n=11) vs. SpHC (n=11)	Species	Shannon	3.40 (1.26)	2.71 (1.01)	0.22	0.22
rHC (n=12) vs. SpHC (n=11)	Phylum	Simpson	0.46 (0.21)	0.42 (0.17)	0.99	0.99
rHC (n=11) vs. SpHC (n=11)	Genus	Simpson	0.61 (0.26)	0.57 (0.20)	0.99	0.99
rHC (n=11) vs. SpHC (n=11)	Species	Simpson	0.71 (0.23)	0.63 (0.20)	0.99	0.99
rHC (n=12) vs. SpHC (n=11)	Phylum	Richness	8.25 (3.22)	7.36 (2.58)	0.38	0.38
rHC (n=11) vs. SpHC (n=11)	Genus	Richness	91.81 (33.43)	79.45 (44.05)	0.37	0.37
rHC (n=11) vs. SpHC (n=11)	Species	Richness	168 (61.89)	131.22 (65.38)	0.12	0.12
rHC (n=12) vs. SpHC (n=11)	Phylum	Evenness	0.39 (0.15)	0.39 (0.15)	0.71	0.71
rHC (n=11) vs. SpHC (n=11)	Genus	Evenness	0.377 (0.17)	0.35 (0.12)	0.67	0.67
rHC (n=11) vs. SpHC (n=11)	Species	Evenness	0.46 (0.15)	0.38 (0.12)	0.25	0.25

Alpha diversity values of Shannon index, Simpson index, richness, and evenness at three taxonomic levels. rHC (n=17) and SpHC (n=11) subjects. Rarefaction levels at 5,000 sequences per sample. The rHC and SpHC mean index score and standard deviation (SD) are displayed above. *Please note that five rHC samples at the phylum taxonomic level, and six rHC samples at the genus and species taxonomic levels were removed. This was due to insufficient sequencing depth, leaving 12 rHC samples at the phylum taxonomic level and 11 rHC samples at the genus and species taxonomic level for final analysis.*

Supplementary Table 2. Differences in nasal microbial community structure between PD patients and controls as assessed by analysis of similarity (ANOSIM).

	Phylum		Genus		Species	
**Nasal Comparisons**	**R value**	**p value**	**R value**	**p value**	**R value**	**p value**
SpHC (n=11) vs. rHC (n=17)	0.079	0.108	0.135	**0.028**	0.214	**0.001**
PD (n=30) vs. rHC (n=17)	0.042	0.917	0.205	**0.002**	0.297	**0.001**
SpPD (n=11) vs. SpHC (n=11)	-0.002	0.420	0.055	0.150	-0.022	0.600

ANOSIM results are based on standardized, and square-root transformed multi-amplicon (V1-V9) sequence data at the phylum, genus, and species taxonomic levels. Significant p values ($p < 0.05$: bold/grey).

Supplementary Table 3. Nasal microbial community structure differences between PD patients and controls as assessed by Permutational Multivariate Analyses of Variance (PERMANOVA).

	Phylum			Genus			Species		
**Nasal Comparisons**	**Pseudo-F**	**p value**	**q value**	**Pseudo-F**	**p value**	**q value**	**Pseudo-F**	**p value**	**q value**
SpHC (n=11) vs. rHC (n=12)	3.179	0.062	0.062	1.056	0.379	0.379	1.156	0.295	0.295
PD (n=30) vs. rHC (n=12)	2.217	0.112	0.112	1.319	0.238	0.238	0.278	**0.028**	**0.028**
SpPD (n=11) vs. SpHC (n=11)	0.468	0.635	0.635	1.353	0.239	0.239	0.417	0.855	0.855

PERMANOVA results are based on Bray-Curtis distances for the multi-amplicon (V1-V9) sequence data at the phylum, genus, and species taxonomic levels. Significance values are based on 9,999 permutations and corrected for multiple testing using the Benjamini-Hochberg method ($q < 0.05$). Significant FDR-P values ($q < 0.05$) are bold/grey.

Supplementary Table 4. Nasal microbial community structure differences between PD patients and controls as assessed by Permutational Analyses of Multivariate Dispersions (PERMDISP).

	Phylum			Genus			Species		
**Nasal Comparisons**	**Pseudo-F**	**p value**	**q value**	**Pseudo-F**	**p value**	**q value**	**Pseudo-F**	**p value**	**q value**
SpHC (n=11) vs. rHC (n=17)	4.487	**0.030**	**0.030**	1.689	0.203	0.203	0.257	0.871	0.871
PD (n=30) vs. rHC (n=17)	0.0009	0.974	0.974	0.012	0.910	0.910	1.135	0.299	0.299
SpPD (n=11) vs. SpHC (n=11)	0.620	0.434	0.434	0.044	0.834	0.834	0.021	0.869	0.869

PERMDISP results are based on Bray-Curtis distances for the multi-amplicon (V1-V9) sequence data at the phylum, genus, and species taxonomic levels. Significance values are based on 9,999 permutations and corrected for multiple testing using the Benjamini-Hochberg method ($q < 0.05$). Significant FDR-P values ($q < 0.05$) are bold/grey.

Supplementary Table 5. The mean relative abundance values between random non-household (rHC) and spousal household (SpHC) healthy controls subjects.

Taxonomic Level	rHC: Mean RA % ± (SD)	SpHC: Mean RA % ± (SD)
**Genus**		
Corynebacterium	30.46 (25.35)	47.77 (29.69)
Moraxella	0.45 (1.19)	0.7 (2.3)
Staphylococcus	13.87 (22.45)	11.13 (13.27)
Alloiococcus	7.06 (17.3)	1.82 (3.66)
Propionibacterium	15.48 (13.74)	18.61 (21.53)
Peptoniphilus	4.00 (6.94)	2.54 (3.02)
Anaerococcus	3.09 (3.44)	3.93 (3.62)
Finegoldia	1.57 (2.92)	2.42 (2.47)
**Species**		
Corynebacterium accolens	24.96 (29.45)	30.44 (29.9)
Corynebacterium propinquum	4.88 (12.41)	15.16 (25.91)
Propionibacterium acnes	17.92 (15.62)	20.72 (22.49)
Moraxella catarrhalis	0.33 (0.91)	0.21 (0.68)
Staphylococcus aureus	4.18 (10.8)	3.22 (6.85)
Peptoniphilus asaccharolyticus	4.16 (8.22)	3.27 (4.32)
Staphylococcus epidermidis	1.53 (1.67)	4.61 (3.59)
Corynebacterium striatum	2.12 (2.37)	2.9 (4.32)
Ralstonia insidiosa	1.19 (1.74)	0.10 (0.13)
Staphylococcus haemolyticus	1.54 (4.24)	1.57 (4.38)
Staphylococcus chromogenes	1.47 (2.91)	0.83 (1.26)
Corynebacterium tuberculostearicum	1.54 (1.50)	1.02 (1.89)
Peptostreptococcus stomatis	0.76 (3.02)	0.01 (0.02)

Deep nasal microbiota of rHC (n=17) and SpHC (n=11) subjects. Mean RA % = average number of sequences per taxa, calculated from the total sum of all sequence counts, depicted as a percentage. Microbial taxa (>1%) shown. (SD) = standard deviation.

Supplementary Table 6. Differential and compositional abundance analysis of the microbial profiles between random non-household (rHC) and spousal household (SpHC) healthy controls subjects, at the genus taxonomic level.

**rHC (n=17) vs. SpHC (n=11)**							
**DESeq2**				**ANCOM**			
**Genus**	**Base Mean**	**Log2 FC (SpHC over rHC)**	**q value**	**Genus**	**Median Percentile Abundances: rHC**	**Median Percentile Abundances: SpHC**	**W Score**
Dermabacter	5.54	5.939	**0.002**	*Acinetobacter*	25.3	200.8	**121**
Delftia	4.84	2.983	**0.006**	**Genera trending towards significance**			
Pelomonas	41.64	-2.315	**0.003**	*Desulfuromusa*	1.0	5.7	89
Serratia	19.15	-2.622	**0.003**	***Burkholderia***	417.0	33.8	70
Ralstonia	78.94	-3.052	**2.544E-04**	***Delftia***	1.7	10.7	44
Lactobacillus	13.84	-3.208	**0.002**				
Blautia	18.58	-3.45	**4.983E-04**				
Bacillus	20.43	-3.559	**4.983E-04**				
Oscillospira	7.17	-3.809	**0.006**				
Burkholderia	290.99	-3.929	**1.640E-14**				
Escherichia	23.67	-4.753	**2.685E-04**				
Paucibacter	8.84	-5.098	**1.111E-05**				
Limnohabitans	7.89	-5.138	**0.001**				
Myroides	4.84	-5.22	**4.997E-04**				
Lentibacillus	1.59	-5.671	**0.003**				
Geothrix	1.98	-5.703	**0.005**				
Curvibacter	11.08	-6.79	**2.119E-05**				
Salinispora	8.6	-7.745	**1.199E-05**				

Genera differential and compositional abundance microbiota differences using DESeq2 and ANCOM. Base Mean = mean of normalized samples. Log2 FC = Log2 fold change of taxa in SpHC compared to rHC subjects. Reference the Methods for DeSeq2 filter details. Median percentile abundance (average of the 25th, 50th, 75th percentiles) and analysis W scores determined using ANCOM. One bacterial genus was significantly identified using ANCOM [filter included removing any sample that was less than 5,000 sequences; any genera that was present in < 5 samples; and whose count across all samples was < 50]. DeSeq2 and ANCOM were corrected for multiple testing using the Benjamini-Hochberg method (FDR-P < 0.05: bold/grey). Taxa identified by both DESeq2 and ANCOM are bolded.

Supplementary Table 7. Differential and compositional abundance analysis of the microbial profiles between random non-household (rHC) and spousal household (SpHC) healthy controls subjects, at the species taxonomic level.

rHC (n=17) vs. SpHC (n=11)							
**DESeq2**				**ANCOM**			
**Species**	**Base Mean**	**Log2 FC (SpHC over rHC)**	**q value**	**Species**	**Median Percentile Abundance: rHC**	**Median Percentile Abundance: SpHC**	**W Score**
Pseudomonas mandelii	4.45	6.029	**2.83E-07**	***Burkholderia xenovorans***	110.8	1.0	**256**
Corynebacterium imitans	2.55	5.858	**0.008**	*Acinetobacter guillouiae*	3.8	116.3	**239**
Anaerococcus hydrogenalis	1.94	5.606	**0.001**	**Species trending towards significance**			
Brevibacterium paucivorans	0.92	5.001	**0.007**	***Burkholderia kururiensis***	43.5	1.0	193
Pseudomonas brenneri	2.4	3.449	**0.007**	***Pseudomonas mandelii***	1.0	8.5	123
Micrococcus yunnanensis	3.25	3.267	**0.008**	*Acinetobacter tjernbergiae*	2.2	13.8	106
Delftia tsuruhatensis	3.11	3.093	**0.008**	*Agromyces succinolyticus*	52.0	1.2	93
Staphylococcus epidermidis	573.22	2.578	**8.34E-06**	***Staphylococcus epidermidis***	250.3	1107.5	68
Streptomyces nanchangensis	9.34	2.459	**0.008**				
Microbacterium paludicola	2.15	-2.716	**0.002**				
Serratia entomophila	19.62	-2.766	**0.002**				
Pelomonas saccharophila	48.06	-2.864	**4.86E-04**				
Corynebacterium acetoacidophilum	3.82	-3.037	**0.01**				
Ralstonia insidiosa	82.01	-3.038	**0.001**				
Burkholderia ubonensis	58.31	-3.239	**1.87E-05**				
Burkholderia vietnamiensis	22.13	-3.266	**3.88E-04**				
Proteus hauseri	3.92	-3.532	**0.008**				
Erythrobacter aquimaris	1.51	-3.817	**0.008**				
Burkholderia phytofirmans	47.08	-4.613	**1.20E-06**				
Escherichia albertii	20.8	-4.833	**7.57E-05**				
Segetibacter aerophilus	0.73	-4.876	**0.008**				
Blautia wexlerae	7.07	-5	**0.002**				
Rhodococcus imtechensis	2.23	-5.028	**0.005**				
Streptomyces danangensis	0.74	-5.132	**0.004**				
Yersinia massiliensis	2.26	-5.218	**0.002**				
Brevundimonas terrae	2.91	-5.419	**0.005**				
Trabulsiella odontotermitis	1.77	-5.644	**0.009**				
Lentibacillus kapialis	1.41	-5.731	**0.003**				
Burkholderia terrae	1.95	-5.782	**0.004**				
Myroides odoratus	5.82	-5.82	**2.10E-04**				
Burkholderia sabiae	2.21	-6.152	**0.002**				
Ralstonia detusculanense	2.27	-6.273	**3.88E-04**				
Dyella ginsengisoli	2.91	-6.524	**3.88E-04**				
Burkholderia phenoliruptrix	13.56	-6.653	**1.42E-06**				
Burkholderia brasilensis	6.49	-6.678	**3.04E-05**				
Burkholderia bryophila	3.52	-6.85	**6.13E-05**				
Curvibacter lanceolatus	10.98	-7.006	**1.23E-05**				

Burkholderia tuberum	6.14	-7.566	**6.97E-06**				
Salinispora tropica	8.97	-8.021	**2.24E-06**				
Burkholderia kururiensis	41.98	-10.154	**2.75E-11**				
Burkholderia xenovorans	101.37	-10.426	**1.29E-17**				

Species differential and compositional abundance microbiota differences using DESeq2 and ANCOM. Base Mean = mean of normalized samples. Log2 FC = Log2 fold change of taxa in SpHC compared to rHC subjects. Reference the Methods for DeSeq2 filter details. Median percentile abundance (average of the 25th, 50th, 75th percentiles) and analysis W scores determined using ANCOM. Two bacterial species were significantly identified using ANCOM [filter included removing any sample that was less than 5,000 sequences; any species that was present in < 5 samples; and whose count across all samples was < 50]. DeSeq2 and ANCOM were corrected for multiple testing using the Benjamini-Hochberg method (FDR-P < 0.05: bold/grey). Taxa identified by both DESeq2 and ANCOM are bolded.

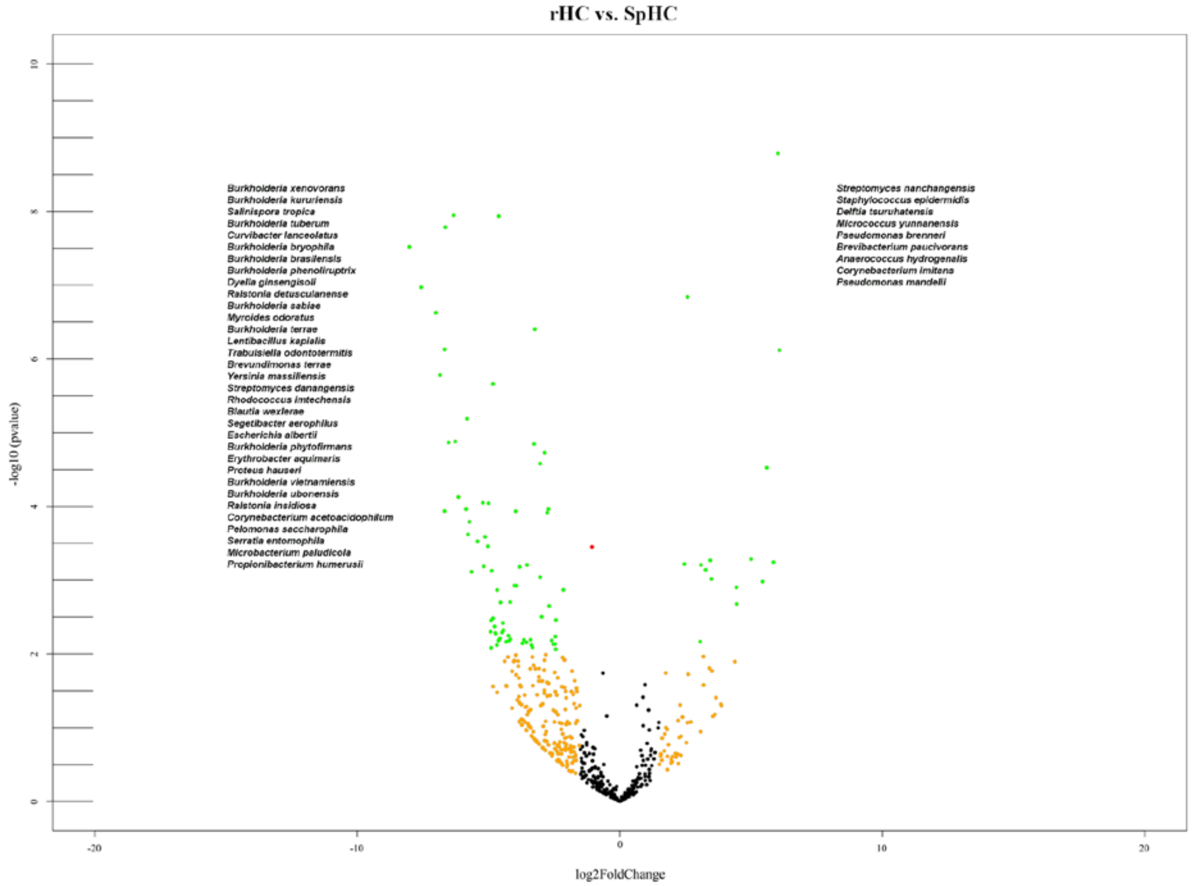

Supplementary Figure 1. Nasal microbial differences between rHC and SpHC subjects. Differential abundance of microbial species measured using DESeq2 within SpHC (n=11) when compared to rHC (n=17) subjects. Volcano plot displays species that had a log2 fold change (> ± 1.5) and FDR-P significant (q < 0.05) (Green); log2 fold change (> ± 1.5) and FDR-P non-significant (q > 0.05) (Red); log2 fold change (> ± 1.5), P-value significant (P < 0.05), and FDR-P value non-significant (q > 0.05) (orange); and log2 fold change (< 1.5), and both P- and FDR-P values are both non-significant (p/q > 0.05) (Black). Based on our stringent threshold (log2 fold change > ± 1.2 and FDR-P < 0.01), 41 bacterial species were differentially abundant between rHC and SpHC subjects. Bacterial species abundances that significantly decreased (32) in SpHC compared to rHC subjects are listed on the left of the volcano plot. Bacterial species abundances that significantly increased (9) in SpHC compared to rHC subjects are listed on the right of the volcano plot.

Supplementary Table 8. Alpha diversity values random non-household (rHC) healthy control and PD subjects.

Comparison Groups	Taxonomic Level	Diversity Indices	rHC: Mean (SD)	PD: Mean (SD)	p value	q value
rHC (n=12) vs. PD (n=30)	Phylum	Shannon	1.184 (0.51)	1.066 (0.40)	0.34	0.34
rHC (n=11) vs. PD (n=30)	Genus	Shannon	2.486 (1.21)	2.060 (0.84)	0.29	0.29
rHC (n=11) vs. PD (n=30)	Species	Shannon	3.405 (1.26)	2.720 (0.94)	0.11	0.11
rHC (n=12) vs. PD (n=30)	Phylum	Simpson	0.460 (0.21)	0.426 (0.18)	0.99	0.99
rHC (n=11) vs. PD (n=30)	Genus	Simpson	0.617 (0.26)	0.572 (0.14)	0.99	0.99
rHC (n=11) vs. PD (n=30)	Species	Simpson	0.711 (0.23)	0.658 (0.13)	0.99	0.99
rHC (n=12) vs. PD (n=30)	Phylum	Richness	8.25 (3.22)	7.066 (2.33)	0.23	0.23
rHC (n=11) vs. PD (n=30)	Genus	Richness	91.818 (33.43)	75.867 (53.59)	0.15	0.15
rHC (n=11) vs. PD (n=30)	Species	Richness	168 (61.89)	122.233 (85.81)	**0.02**	**0.02**
rHC (n=12) vs. PD (n=30)	Phylum	Evenness	0.391 (0.15)	0.389 (0.15)	0.54	0.54
rHC (n=11) vs. PD (n=30)	Genus	Evenness	0.3771 (0.17)	0.329 (0.08)	0.36	0.36
rHC (n=11) vs. PD (n=30)	Species	Evenness	0.460 (0.15)	0.393 (0.09)	0.18	0.18

Alpha diversity values of Shannon index, Simpson index, richness, and evenness at three taxonomic levels. rHC (n=17) and PD (n=30) subjects. Rarefaction levels at 5,000 sequences per sample. The rHC and PD mean index score and standard deviation (SD) are displayed above. Significant p-values ($P<0.05$) and FDR P values ($q < 0.05$: bold/grey). *Please note that five rHC samples at the phylum taxonomic level, and six rHC samples at the genus and species taxonomic levels were removed. This was due to insufficient sequencing depth, leaving 12 rHC samples at the phylum taxonomic level and 11 rHC samples at the genus and species taxonomic level for final analysis.*

Supplementary Table 9. The mean relative abundances values of the microbial profiles between random non-household (rHC) healthy control and PD subjects.

Taxonomic Level	rHC: Mean RA % ± (SD)	PD: Mean RA % ± (SD)
**Genus**		
Corynebacterium	30.46 (25.35)	38.9 (28.54)
Moraxella	0.45 (1.19)	8.55 (26.51)
Staphylococcus	13.87 (22.45)	3.15 (10.74)
Alloiococcus	7.06 (17.3)	13.47 (16.1)
Propionibacterium	15.48 (13.74)	14.88 (14.11)
Peptoniphilus	4.00 (6.94)	1.96 (2.93)
Anaerococcus	3.09 (3.44)	1.79 (4.51)
Finegoldia	1.57 (2.92)	1.01 (2.66)
**Species**		
Corynebacterium accolens	24.96 (29.45)	22.65 (24.74)
Corynebacterium propinquum	4.88 (12.41)	14.48 (23.28)
Propionibacterium acnes	17.92 (15.62)	17.92 (15.5)
Moraxella catarrhalis	0.33 (0.91)	8.79 (21.84)
Staphylococcus aureus	4.18 (10.8)	1.66 (2.83)
Peptoniphilus asaccharolyticus	4.16 (8.22)	2.12 (3.48)
Staphylococcus epidermidis	1.53 (1.67)	4.08 (5.89)
Corynebacterium striatum	2.12 (2.37)	4.37 (8.12)
Ralstonia insidiosa	1.19 (1.74)	6.92 (15.77)
Staphylococcus haemolyticus	1.54 (4.24)	0.61 (1.24)
Staphylococcus chromogenes	1.47 (2.91)	0.56 (0.71)
Corynebacterium tuberculostearicum	1.54 (1.50)	1.34 (2.59)
Peptostreptococcus stomatis	0.76 (3.02)	0.01 (0.03)

Deep nasal microbiota of rHC (n=17) and PD (n=30) subject. Mean RA % = average number of sequences per taxa, calculated from the total sum of all sequence counts, depicted as a percentage. Microbial taxa (>1%) shown. (SD) = standard deviation.

Supplementary Table 10. Differential and compositional abundance analysis of the microbial profiles between random non-household (rHC) healthy control and PD subjects, at the genus taxonomic level.

**rHC (n=17) vs. PD (n=30)**							
**DESeq2**				**ANCOM**			
**Genus**	**Base Mean**	**Log2 FC (PD over rHC)**	**q value**	**Genus**	**Median Percentile Abundance: rHC**	**Median Percentile Abundance: PD**	**W Score**
Moraxella	7927.57	7.869	**7.840E-10**	***Acinetobacter***	25.3	148.2	**177**
Methylophaga	5.17	4.908	**0.004**	***Desulfuromusa***	1.0	5.3	**160**
Dermabacter	2.72	4.538	**0.001**	**Genera trending towards significance**			
Delftia	8.98	3.283	**1.970E-04**	***Curvibacter***	16.7	1.0	139
Ralstonia	1165.2	3.261	**0.004**	***Paucibacter***	15.2	1.5	114
Desulfuromusa	6.47	2.947	**0.008**	***Delftia***	1.7	10.2	89
Acinetobacter	130.51	1.545	**0.015**	***Burkholderia***	417.0	56.0	55
Dietzia	33.18	-1.967	**0.009**				
Burkholderia	328.82	-2.046	**0.006**				
Pelomonas	38.18	-2.335	**1.621E-04**				
Listeria	5.09	-2.651	**0.002**				
Serratia	29.92	-2.728	**0.001**				
Bacillus	18.31	-2.812	**0.001**				
Yersinia	38.68	-2.836	**7.078E-06**				
Erythrobacter	1.52	-2.984	**0.003**				
Alkaliphilus	4.49	-2.988	**4.613E-06**				
Lactobacillus	20.32	-3.042	**3.948E-04**				
Lachnospira	3.13	-3.2	**0.003**				
Rhodothermus	0.97	-3.505	**0.005**				
Klebsiella	13.21	-3.701	**0.001**				
Johnsonella	1.25	-4.149	**0.003**				
Paucibacter	7.89	-4.251	**9.790E-08**				
Trabulsiella	17.64	-4.306	**0.001**				
Dechloromonas	0.7	-4.347	**0.002**				
Aminiphilus	0.33	-4.588	**0.002**				
Thalassospira	1.27	-4.913	**0.001**				
Limnohabitans	6.35	-5.091	**5.061E-05**				
Edwardsiella	1.1	-5.465	**0.005**				
Turicibacter	2.04	-5.486	**0.002**				
Gemmatimonas	1.32	-5.574	**0.006**				
Geothrix	1.61	-6.344	**1.701E-05**				
Curvibacter	9.07	-6.581	**1.253E-08**				

Genera differential and compositional abundance microbiota differences using DESeq2 and ANCOM. Base Mean = mean of normalized samples. Log2 FC = Log2 fold change of taxa in PD compared to rHC subjects. Reference the Methods for DeSeq2 filter details. Median percentile abundance (average of the 25th, 50th, 75th percentiles) and analysis W scores determined using ANCOM. Two bacterial genera were significantly identified using ANCOM [filter included removing any sample that was less than 5,000 sequences; any genera that was present in < 5 samples; and whose count across all samples was < 50]. DeSeq2 and ANCOM were corrected for multiple testing using the Benjamini-Hochberg method (FDR-P < 0.05: bold/grey). Taxa identified by both DESeq2 and ANCOM are bolded above.

Supplementary Table 11. Differential and compositional abundance analysis of the microbial profiles between random non-household (rHC) healthy control and PD subjects, at the species taxonomic level.

rHC (n=17) vs. PD (n=30)							
**DESeq2**				**ANCOM**			
**Species**	**Base Mean**	**Log2FC (PD over rHC)**	**q value**	**Species**	**Median Percentile Abundance: rHC**	**Median Percentile Abundance: PD**	**W Score**
Pseudomonas mandelii	8.12	6.249	**1.224E-09**	*Acinetobacter guillouiae*	3.8	64.3	**290**
Moraxella catarrhalis	776.37	5.671	**1.292E-04**	**Species trending towards significance**			
Anaerococcus hydrogenalis	3.51	5.458	**0.001**	***Pseudomonas mandelii***	1.0	10.0	231
Pseudomonas brenneri	6.43	3.764	**2.928E-04**	*Burkholderia xenovorans*	110.8	4.2	152
Delftia tsuruhatensis	5.86	3.546	**0.001**	***Curvibacter lanceolatus***	11.8	1.0	152
Micrococcus yunnanensis	4.87	3.319	**0.002**	***Pseudomonas brenneri***	1.0	6.2	129
Ralstonia insidiosa	960.13	3.304	**0.002**	***Corynebacterium atypicum***	55.2	3.8	118
Corynebacterium propinquum	3574.63	2.954	**0.003**	***Burkholderia phenoliruptrix***	15.5	1.0	113
Staphylococcus epidermidis	420.28	1.693	**2.463E-06**	***Corynebacterium ulceribovis***	39.0	2.6	108
Propionibacterium humerusii	120.94	-1.402	**1.628E-04**	*Acinetobacter gerneri*	1.5	10.6	94
Kocuria rosea	8.57	-1.84	**0.009**	*Acinetobacter tjernbergiae*	2.2	7.3	80
Corynebacterium pyruviciproducens	2.37	-1.852	**0.010**	***Peptoniphilus methioninivorax***	30.5	2.0	79
Microbacterium paludicola	1.61	-2.283	**0.004**	***Corynebacterium acetoacidophilum***	13.7	1.3	71
Burkholderia multivorans	5.86	-2.344	**0.009**	***Kocuria rosea***	45.0	5.0	64
Pelomonas saccharophila	35.76	-2.471	**1.750E-04**				
Haemophilus parainfluenzae	45.63	-2.53	**0.001**				
Burkholderia vietnamiensis	18.94	-2.572	**2.117E-04**				
Corynebacterium doosanense	1.41	-2.667	**0.007**				
Corynebacterium atypicum	7.08	-2.768	**5.043E-05**				
Corynebacterium ulceribovis	8.35	-2.884	**1.514E-04**				
Burkholderia ubonensis	44.8	-2.895	**2.842E-06**				
Peptoniphilus methioninivorax	6.12	-2.986	**1.628E-04**				
Escherichia albertii	19.17	-3.048	**0.010**				
Tolumonas auensis	2.04	-3.086	**0.004**				
Staphylococcus hyicus	1.23	-3.107	**0.001**				
Corynebacterium acetoacidophilum	2.65	-3.181	**2.928E-04**				
Myroides odoratus	4.16	-3.261	**0.004**				
Corynebacterium massiliense	1.7	-3.385	**0.001**				
Erythrobacter aquimaris	1.05	-3.394	**1.280E-04**				
Actinomyces naturae	1.68	-3.423	**1.628E-04**				
Johnsonella ignava	0.83	-3.439	**0.008**				
Streptomyces danangensis	0.55	-3.46	**0.001**				
Proteus hauseri	2.9	-3.513	**0.001**				
Peptoniphilus tyrrelliae	1.76	-3.644	**0.003**				
Lachnospira pectinoschiza	3.29	-3.655	**0.001**				
Mannheimia caviae	13.15	-3.687	**1.505E-04**				
Peptoniphilus olsenii	2.66	-3.736	**0.001**				
Azospirillum palatum	1.51	-3.742	**0.001**				

Campylobacter canadensis	0.39	-3.743	**0.001**				
Actinomyces meyeri	1.87	-3.78	**0.001**				
Curvibacter gracilis	0.62	-4.05	**0.010**				
Stenotrophomonas pavanii	0.71	-4.076	**0.004**				
Burkholderia mallei	2.9	-4.292	**0.001**				
Burkholderia bryophila	2.46	-4.303	**1.280E-04**				
Dietzia kunjamensis	1.74	-4.348	**1.862E-05**				
Rhodothermus clarus	1.14	-4.423	**1.278E-04**				
Yersinia pestis	0.39	-4.519	**0.004**				
Selenomonas flueggei	0.28	-4.558	**0.002**				
Mycobacterium chitae	0.33	-4.627	**3.878E-04**				
Aminiphilus circumscriptus	0.32	-4.703	**4.098E-04**				
Actinobacillus rossii	0.62	-4.977	**2.928E-04**				
Geothrix fermentans	0.62	-5.015	**0.004**				
Burkholderia brasilensis	4.13	-5.119	**2.463E-06**				
Blautia wexlerae	4.91	-5.177	**1.491E-06**				
Burkholderia phenoliruptrix	9.04	-5.399	**6.088E-07**				
Bacteroides denticanum	0.95	-5.494	**0.002**				
Parabacteroides goldsteinii	1.61	-5.549	**0.002**				
Yersinia massiliensis	1.59	-5.816	**6.088E-07**				
Curvibacter lanceolatus	6.89	-7.458	**4.799E-10**				

Species differential and compositional abundance microbiota differences using DESeq2 and ANCOM. Base Mean = mean of normalized samples. Log2 FC = Log2 fold change of taxa in PD compared to rHC subjects. Reference the Methods for DeSeq2 filter details. Median percentile abundance (average of the 25th, 50th, 75th percentiles) and analysis W scores determined using ANCOM. One bacterial species was significantly identified using ANCOM [filter included removing any sample that was less than 5,000 sequences; any species that was present in < 5 samples; and whose count across all samples was < 50]. DeSeq2 and ANCOM were corrected for multiple testing using the Benjamini-Hochberg method (FDR-P < 0.05: bold/grey). Taxa identified by both DESeq2 and ANCOM are bolded above.

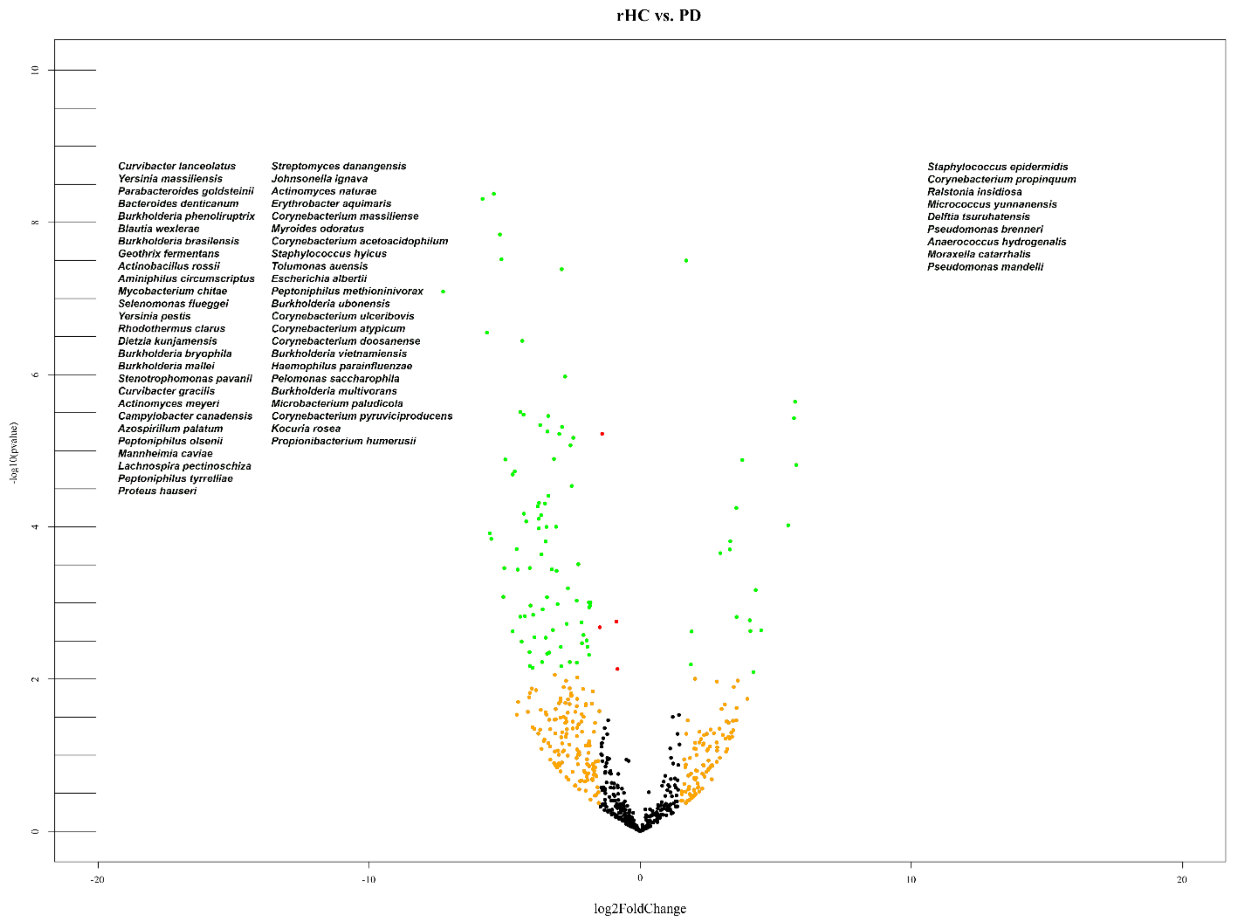

Supplementary Figure 2. Nasal microbial differences between rHC and PD subjects. Differential abundance differences were measured using DESeq2 within PD (n=30) subjects in comparison to rHC (n=17) subjects. Volcano plot displays species that had a log2 fold change (> ± 1.5) and FDR-P significant (q < 0.05) (Green); log2 fold change (> ± 1.5) and FDR-P non-significant (P > 0.05) (Red); log2 fold change (> ± 1.5), P-value significant (P < 0.05), and FDR-P value non-significant (P > 0.05) (orange); and log2 fold change (< ± 1.5) and both P- and FDR-P values are both non-significant (p/q > 0.05) (Black). Based on our stringent threshold (log2 fold change > ± 1.2 and FDR P < 0.01), there were a 59 bacterial species significantly differentially abundant between rHC and PD subjects. Bacterial species abundances that significantly decreased (50) in PD compared to rHC subjects are listed on the left of the volcano plot. Bacterial species abundances that significantly increased (9) in PD compared to rHC subjects are listed on the right of the volcano plot.

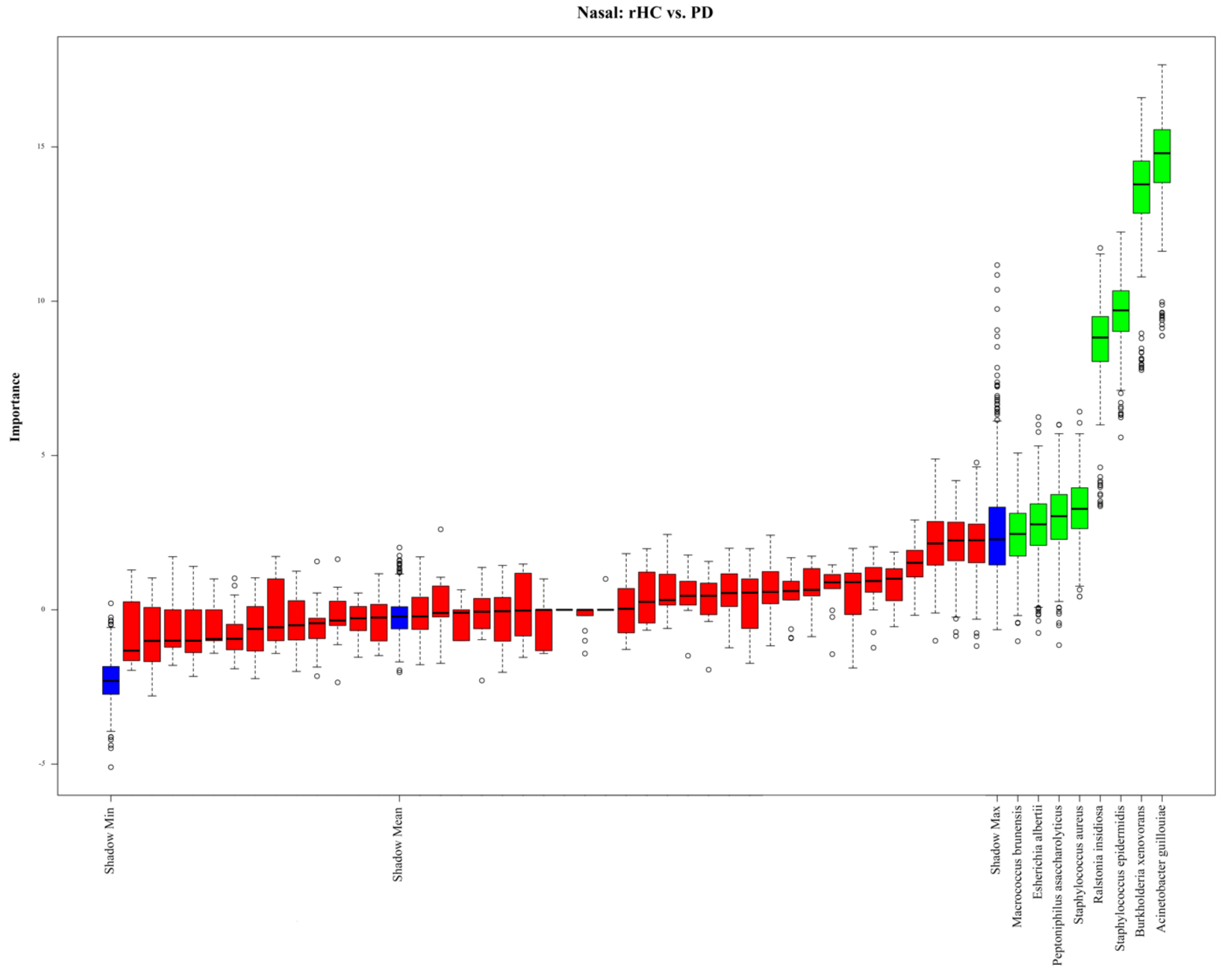

Supplementary Figure 3. Features identified in PD subjects. Predictive model based on the species-level abundance in PD subjects vs. rHC subjects using Boruta. Green boxes are bacterial species that are associated with driving PD microbiome differences from rHC were measured using Boruta feature selection algorithm. Blue boxes correspond to minimal, average, and maximum Z score of the shadow species, which are shuffled version of real species introduced to Random Forest classifier and act as benchmarks to detect truly predictive species. Red boxes are bacterial species that were not associated with driving differences in PD subjects were less than the shadow max.

Supplementary Table 12. Alpha diversity values of spousal household (SpHC) healthy controls and household PD (SpPD) subjects.

**Comparison Groups**	**Taxonomic Level**	**Diversity Indices**	**SpHC: Mean (SD)**	**SpPD: Mean (SD)**	**p value**	**q value**
SpHC (n=11) vs. SpPD (n=11)	Phylum	Shannon	1.114 (0.45)	1.057 (0.30)	0.82	0.82
SpHC (n=11) vs. SpPD (n=11)	Genus	Shannon	2.227 (0.89)	2.027 (0.84)	0.62	0.62
SpHC (n=11) vs. SpPD (n=11)	Species	Shannon	2.719 (1.01)	2.678 (0.94)	0.82	0.82
SpHC (n=11) vs. SpPD (n=11)	Phylum	Simpson	0.427 (0.17)	0.413 (0.13)	0.99	0.99
SpHC (n=11) vs. SpPD (n=11)	Genus	Simpson	0.577 (0.20)	0.558 (0.14)	0.99	0.99
SpHC (n=11) vs. SpPD (n=11)	Species	Simpson	0.633 (0.20)	0.654 (0.13)	0.99	0.99
SpHC (n=11) vs. SpPD (n=11)	Phylum	Richness	7.363 (2.58)	7.182 (2.82)	0.78	0.78
SpHC (n=11) vs. SpPD (n=11)	Genus	Richness	79.454 (44.05)	75.363 (53.58)	0.58	0.58
SpHC (n=11) vs. SpPD (n=11)	Species	Richness	131.181 (65.38)	126.909 (85.81)	0.41	0.41
SpHC (n=11) vs. SpPD (n=11)	Phylum	Evenness	0.395 (0.15)	0.382 (0.07)	0.92	0.92
SpHC (n=11) vs. SpPD (n=11)	Genus	Evenness	0.357 (0.12)	0.331 (0.08)	0.62	0.62
SpHC (n=11) vs. SpPD (n=11)	Species	Evenness	0.387 (0.12)	0.388 (0.09)	0.97	0.97

Alpha diversity values of Shannon index, Simpson index, richness, and evenness at three taxonomic levels: phylum, genus, and species within SpHC (n=11) and SpPD (n=11) subjects. Rarefaction levels at 5,000 sequences. SpHC and SpPD mean index scores and standard deviation (SD) are displayed above. Adjusted paired t-test p- and q-values shown.

Supplementary Table 13. The mean relative abundances and corresponding significant differential abundance analysis values of the microbial profiles between spousal household (SpHC) healthy control subjects paired with their corresponding household PD (SpPD) subjects.

Taxonomic Level	Mean RA % ± (SpHC)	Mean RA % ± (SpPD)	p value
**Genus**			
Chromobacterium	0.0038	0.0917	**0.04**
Dermacoccus	0.0045	0.0002	**0.04**
Moraxella	0.1952	14.8382	**0.03**
Staphylococcus	6.7936	1.7203	**< 0.01**
Thermodesulfovibrio	0.0067	0.0013	**0.02**
**Species**			
Anaerococcus prevotii	0.0579	0.0033	**< 0.01**
Corynebacterium bovis	0.0021	0.0933	**< 0.01**
Moraxella catarrhalis	0.0520	5.2996	**< 0.01**
Staphylococcus aureus	1.6760	0.5626	**< 0.01**
Staphylococcus chromogenes	0.4983	0.2102	**< 0.01**
Staphylococcus cohnii	0.0743	0.0237	**< 0.01**
Staphylococcus epidermidis	3.2574	1.1079	**< 0.01**
Streptococcus fryi	0.0128	0.0007	**< 0.01**
Streptococcus infantis	0.0581	0.0080	**< 0.01**
Trabulsiella farmeri	1.1171	0.0000	**< 0.01**
Yersinia frederiksenii	0.0038	0.0027	**< 0.01**

Mean RA % = average relative abundance represented as a percentage. Significant differential abundances were measured using Wilcoxon signed-rank paired test within SpHC (n = 11) subjects compared to SpPD (n = 11) subjects. P-value ($p < 0.05$) shown.

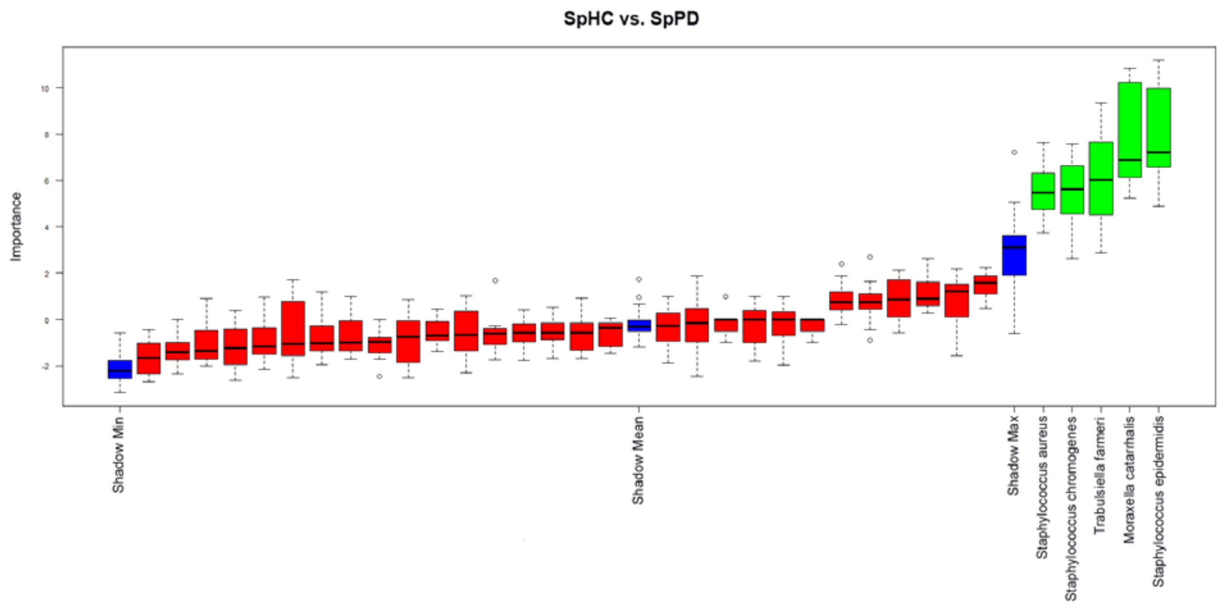

Supplementary Figure 4. Features identified in SpPD subjects. Predictive model based on the species-level abundance in SpPD subjects vs. SpHC subjects using Boruta. Green boxes are bacterial species that are associated with driving PD microbiome differences from rHC were measured using Boruta feature selection algorithm. Blue boxes correspond to minimal, average, and maximum Z score of the shadow species, which are shuffled version of real species introduced to Random Forest classifier and act as benchmarks to detect truly predictive species. Red boxes are bacterial species that were not associated with driving differences in PD subjects were less than the shadow max.

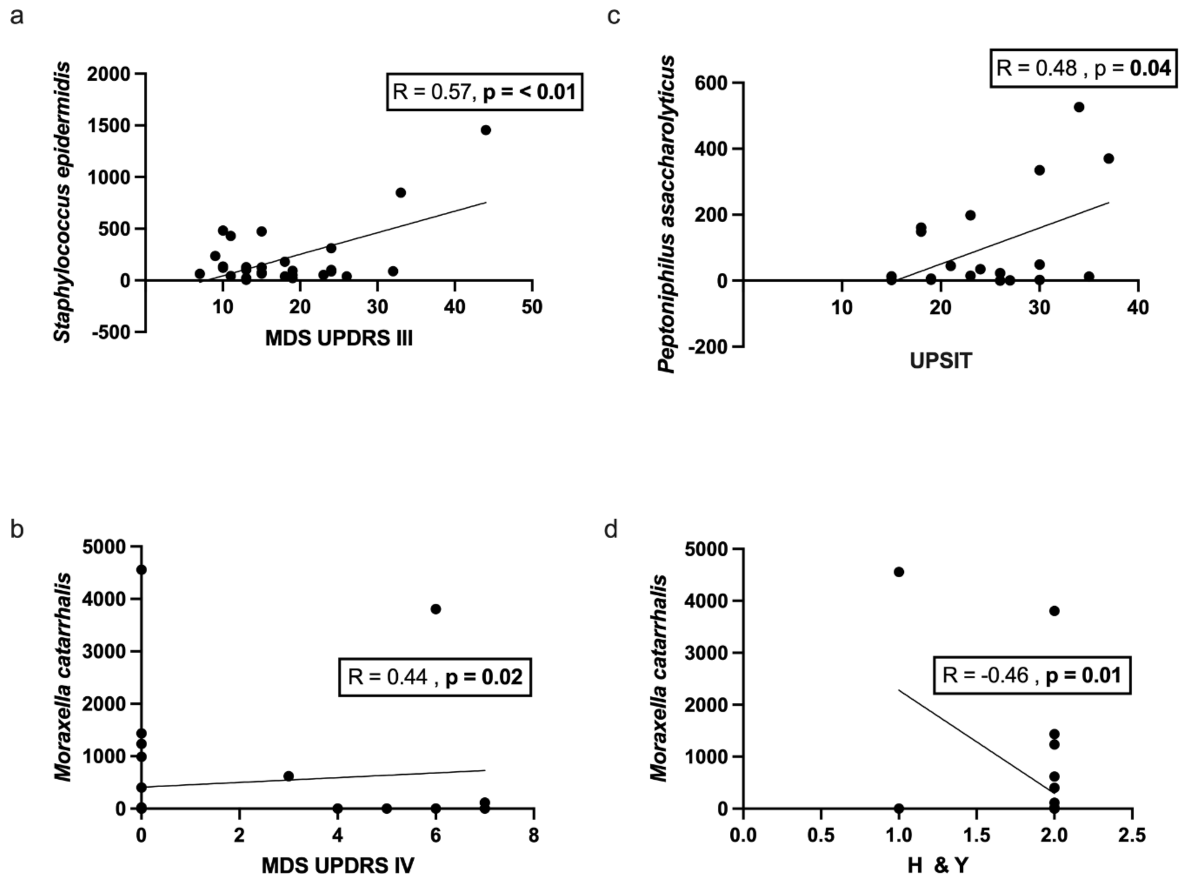

Supplementary Figure 5. **Altered deep nasal microbial community correlated with clinical variables of PD subjects.** (A-D). Clinical characteristic of study PD subjects, including H & Y, MDS-UPDRS III, IV, and UPSIT were correlated with the species taxonomic level of PD subjects' deep nasal microbiome (N = 29) using Pearson's correlation on rarefied data. R = Pearson's correlation coefficient, ranging from values +1 to -1. R-squared value = the square of Pearson's correlation coefficient. Significant P values (P < 0.05).

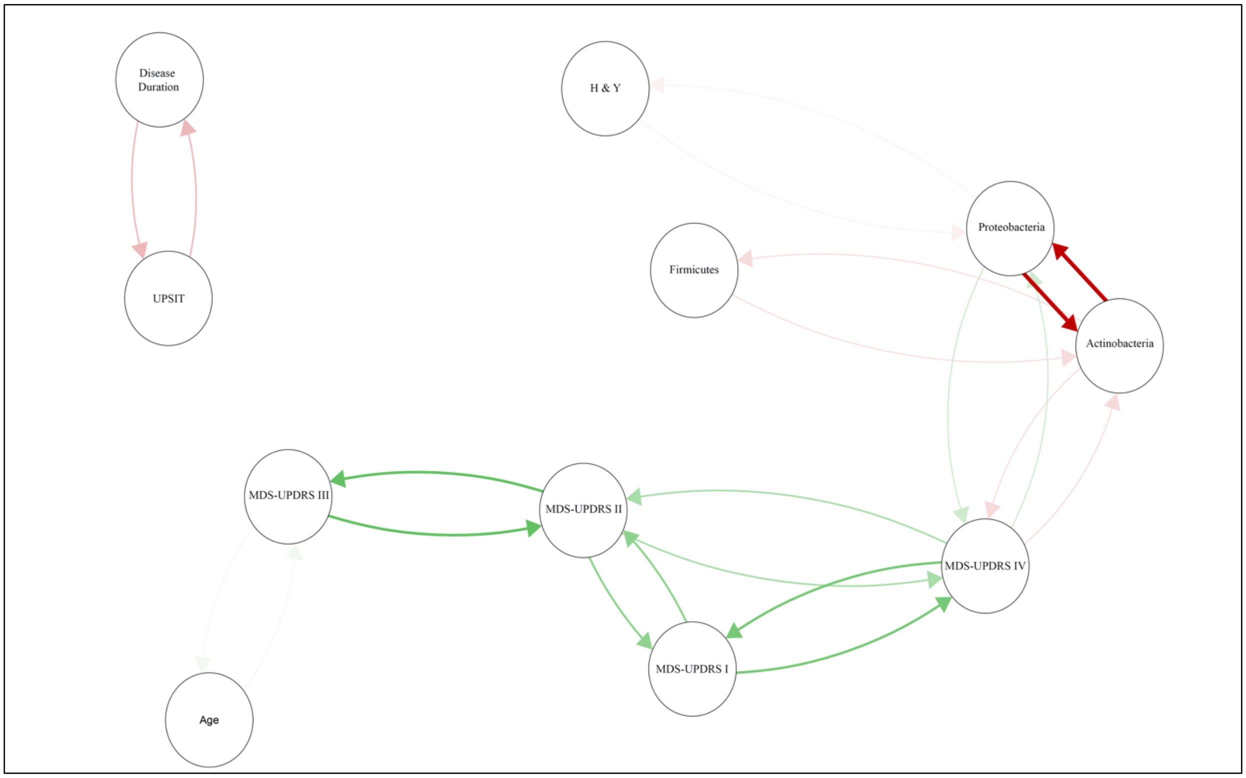

Supplementary Figure 6. **Visualization of multiple associations present in PD patients' clinical features with deep nasal microbiota at the phylum taxonomic level.** (A) PD patient clinical characteristics in correlation with taxa at the phylum taxonomic level. Positive correlations (green arrows, negative correlations (red arrows), strong (thick edges) and weak (thin edges, less saturated) correlations are present between PD patient clinical features and bacterial phyla. Correlation arrows displayed are p value significant ($P < 0.05$) and R value (> 0.3).

16S rRNA V4 Sequencing Analysis

We sought to assess whether the deep nasal sinus cavity microbial genomic composition data identified by sequencing Swift 16S rRNA (V1-V9) multi-amplicons would differ from the microbial composition identified by sequencing analysis using the conventional 16S rRNA variable region 4 (V4) amplicons as a target outcome which was used in prior studies of nasal microbiota compositions in PD. We conducted an additional 16S rRNA (V4) amplicon sequencing on the deep nasal sinus cavity microbial samples of the same PD, rHC, SpHC, and SpPD subjects assessed previously using 16S rRNA (V1-V9) genomic amplicon sequencing. To measure alpha diversity, we rarefied the BIOM to 2,300 sequences per sample and thus removed a total of three samples, due to low sequence counts (1 rHC and 2 SpHC). No significant differences in alpha diversity indices were observed between groups comparisons, at the taxonomic level of genus (**Table S14**).

Of the three group comparisons, the microbial community structure was significantly different only between the rHC and SpHC subjects, at the genus taxonomic level (**Tables S15-16**). Four families and six genera were significantly differentially abundant between rHC and SpHC subjects (**Table S17-18**). Furthermore, the genus *Burkholderia-Caballeronia-Paraburkholderia* (W = 68) in the nasal microbial composition was significantly decreased in abundance in the SpHC compared to the rHC subjects (**Table S18).** Upon examining rHC and PD subjects, eight genera were significantly differentially abundant (**Tables S19-20**). Similar to the multi-amplicon 16S rRNA results, the 16S rRNA V4 dataset identified genus *Moraxella* as significantly increased in PD subjects compared to rHC subjects (FDR-P<0.01). Again, the genus *Burkholderia-Caballeronia-Paraburkholderia* (W = 112) in the nasal microbial composition was significantly decreased in abundance in the PD compared to the rHC subjects

(**Table S20**). When we specifically assessed the differential abundance in taxa within the household subjects (SpPD and SpHC) using a Wilcoxon signed-rank paired test, there were significant differences of three genera, including *Staphylococcus* increased in SpPD compared to SpHC subjects (**Table S21**).

Lastly, Boruta feature selection algorithm identified bacterial genera that are associated with driving changes observed in the deep nasal microbial composition in the PD subjects from the 16S rRNA (V4) genomic analysis. Similar to the Boruta results observed with 16S rRNA (V1-V9) analysis, *Ralstonia*, *Acinetobacter*, and *Burkholderia,* were amongst the genera associated with driving differences in the deep nasal microbiota of PD subjects when compared to rHC subjects (**Figure S6a**). Additionally, when we assessed the spousal household subjects, genus *Staphylococcus* was associated with driving differences observed in the nasal microbiota of spousal PD subjects (**Figure S6b**). Overall, the conventional 16S rRNA (V4) sequencing results further confirmed the changes in the deep nasal microbial composition of PD subjects, as observed in our analysis of the 16S rRNA gene multi-amplicon (V1-V9) sequencing.

Supplementary Table 14. 16S rRNA (V4) alpha-diversity comparisons between healthy controls and PD subjects, at the taxonomic level of genus.

**Comparison Groups**	**Diversity Indices**	**Mean (SD**	**Mean (SD)**	**p value**	**q value**
rHC (n=16) vs. SpHC (n=9)	Shannon	rHC: 2.941 (1.463)	SpHC: 2.856 (0.839)	0.691	0.691
rHC (n=16) vs. SpHC (n=9)	Simpson	rHC: 0.679 (0.281)	SpHC: 0.758 (0.156)	0.734	0.734
rHC (n=16) vs. SpHC (n=9)	Observed Features	rHC: 38.5 (32.193)	SpHC: 34.555 (12.904)	0.733	0.733
rHC (n=16) vs. SpHC (n=9)	Evenness	rHC: 0.568 (0.244)	SpHC: 0.565 (0.147)	0.610	0.610
rHC (n=16) vs. PD (n=30)	Shannon	rHC: 2.941 (1.463)	PD: 2.553 (1.121)	0.310	0.310
rHC (n=16) vs. PD (n=30)	Simpson	rHC: 0.679 (0.281)	PD: 0.666 (0.219)	0.356	0.356
rHC (n=16) vs. PD (n=30)	Observed Features	rHC: 38.5 (32.193)	PD: 39.033 (26.861)	0.510	0.510
rHC (n=16) vs. PD (n=30)	Evenness	rHC: 0.568 (0.244)	PD: 0.499 (0.171)	0.221	0.221
SpHC (n=9) vs. SpPD (n=11)	Shannon	SpHC: 2.856 (0.839)	SpPD: 2.458 (1.371)	0.183	0.183
SpHC (n=9) vs. SpPD (n=11)	Simpson	SpHC: 0.758 (0.156)	SpPD: 0.642 (0.205)	0.183	0.183
SpHC (n=9) vs. SpPD (n=11)	Observed Features	SpHC: 34.555 (12.904)	SpPD: 37.545 (40.721)	0.322	0.322
SpHC (n=9) vs. SpPD (n=11)	Evenness	SpHC: 0.565 (0.147)	SpPD: 0.510 (0.181)	0.382	0.382

Alpha diversity values of Shannon index, Simpson index, Observed features, and Evenness at the genus taxonomic level: rHC (n=17), SpHC (n=11), PD (n=30), and SpPD (n=11). Rarefaction levels at 2,300 sequences. The groups mean index score and standard deviation (SD) are displayed above. . *Please note that one rHC and 2 SpHC samples were removed. This was due to insufficient sequencing depth, leaving 16 rHC samples and 9 SpHC samples for final analysis.*

Supplementary Table 15. 16S rRNA (V4) nasal microbial community structure differences between PD and controls, assessed by PERMANOVA.

	Genus		
**Nasal Comparisons**	**Pseudo-F**	**p**	**q**
SpHC (n=9) vs. rHC (n=16)	1.279	0.203	0.203
PD (n=30) vs. rHC (n=16)	1.529	0.106	0.106
SpPD (n=11) vs. SpHC (n=9)	1.336	0.207	0.207

PERMANOVA results are based on Bray-Curtis distances for the multi-amplicon (V4) sequence data. Significance values are based on 9,999 permutations. The p- and q-values are displayed.

Supplementary Table 16. 16S rRNA (V4) nasal microbial community structure differences between PD and controls, assessed by PERMDISP.

	Genus		
**Nasal Comparisons**	**Pseudo-F**	**p**	**q**
SpHC (n=9) vs. rHC (n=16)	11.394	**0.002**	**0.002**
PD (n=30) vs. rHC (n=16)	2.528	0.118	0.118
SpPD (n=11) vs. SpHC (n=9)	1.993	0.138	0.138

PERMDISP results are based on Bray-Curtis distances for the multi-amplicon (V4) sequence data. Significance values are based on 9,999 permutations. Significant p- and q-values (< 0.05: bold/grey).

Supplementary Table 17. 16S rRNA (V4) mean relative abundance values of the microbial profiles between random non-household (rHC) and spousal household (SpHC) healthy controls subjects.

Taxonomic Level	rHC: Mean RA % ± (SD)	SpHC: Mean RA % ± (SD)
**Family**		
Corynebacteriaceae	32.64 (30.6)	50.6 (22.16)
Staphylococcaceae	17.24 (26.82)	18.93 (21.89)
Peptostreptococcales-Tissierellales	9.96 (18.1)	14.48 (13.35)
Carnobacteriaceae	5.66 (18.15)	2.8 (5.73)
Moraxellaceae	1.72 (5.93)	2.47 (3.89)
Burkholderiaceae	5.69 (9.95)	0.15 (0.3)
Enterobacteriaceae	2.09 (5.14)	1.3 (3.55)
Muribaculaceae	1.81 (5.93)	0.11 (0.35)
Erysipelotrichaceae	1.53 (6.29)	0 (0)
Streptococcaceae	0.74 (1.23)	0.84 (1.29)
Lachnospiraceae	3.64 (6.57)	0.32 (0.63)
Neisseriaceae	0.19 (0.28)	0.48 (1.37)
**Genus**		
Corynebacterium	30.52 (31.18)	48.33 (21.73)
Staphylococcus	17.1 (26.85)	18.93 (21.89)
Dolosigranulum	5.65 (18.15)	2.8 (5.73)
Moraxella	0.23 (0.94)	0.7 (2.33)
Anaerococcus	2.87 (5.21)	6.95 (7.44)
Peptoniphilus	4.13 (7.89)	3.44 (4.06)
Finegoldia	2.01 (4.92)	4.08 (5.08)
Ralstonia	0.81 (1.81)	0.14 (0.3)
Lawsonella	2.12 (4.23)	2.27 (3.52)
Enterobacteriaceae;g_Unclassified	1.84 (4.49)	1.29 (3.52)
Muribaculaceae	1.81 (5.93)	0.11 (0.35)
Dubosiella	1.35 (5.56)	0 (0)
Burkholderia-Caballeronia-Paraburkholderia	4.86 (8.57)	0 (0)
Streptococcus	0.7 (1.24)	0.82 (1.29)

Deep nasal microbiota of rHC (n=17) and SpHC (n=11) subjects. Mean RA % = average number of sequences per taxa, calculated from the total sum of all sequence counts, depicted as a percentage. Microbial taxa (>1%) shown. (SD) = standard deviation.

Supplementary Table 18. 16S rRNA (V4) differential and compositional abundance analysis of the microbial profiles between random non-household (rHC) and spousal household (SpHC) healthy controls subjects, at the genus taxonomic level.

**rHC (n=17) vs. SpHC (n=11)**							
**DESeq2**				**ANCOM**			
**Genus**	**Base Mean**	**Log2 FC (SpHC over rHC)**	**q-value**	**Genus**	**Median Percentile Abundances: rHC**	**Median Percentile Abundances: SpHC**	**W Score**
Comamonas	2.75	5.895	**0.049**	***Burkholderia-Caballeronia-Paraburkholderia***	1,842.0	1.0	**68**
Gemella	1.71	5.575	**0.049**	**Genera trending towards significance**			
Lachnospiraceae Unclassified	166.72	-5.089	**0.012**	*Acinetobacter*	1.0	111.3	44
Faecalibacterium	3.46	-6.518	**0.049**	***Gemella***	1.0	8.7	11
Bacteroides	141.20	-10.670	**1.74E-07**	*Ralstonia*	36.6	1.0	9
Burkholderia-Caballeronia-Paraburkholderia	465.04	-13.174	**4.73E-17**				

Genera differential and compositional abundance microbiota differences using DESeq2 and ANCOM. Base Mean = mean of normalized samples. Log2 FC = Log2 fold change of taxa in SpHC compared to rHC subjects. Reference the Methods for DeSeq2 filter details. Median percentile abundance (average of the 25th, 50th, 75th percentiles) and analysis W scores determined using ANCOM. One bacterial genus was significantly identified using ANCOM [filter included removing any sample that was less than 2,300 sequences; any genus that was present in < 2 samples; and whose count across all samples was < 10]. DeSeq2 and ANCOM were corrected for multiple testing using the Benjamini-Hochberg method (FDR-P < 0.05: bold/grey). Taxa identified by both DESeq2 and ANCOM are bolded.

Supplementary Table 19. 16S rRNA (V4) mean relative abundance values of the microbial profiles in random non-household (rHC) healthy control and PD subjects.

Taxonomic Level	rHC: Mean RA % ± (SD)	PD: Mean RA % ± (SD)
**Family**		
Corynebacteriaceae	32.64 (30.6)	41.96 (29.55)
Staphylococcaceae	17.24 (26.82)	10.41 (14.57)
Peptostreptococcales-Tissierellales	9.96 (18.1)	8.42 (14.34)
Carnobacteriaceae	5.66 (18.15)	10.72 (18.91)
Moraxellaceae	1.72 (5.93)	13.39 (24.89)
Burkholderiaceae	5.69 (9.95)	6.99 (16.05)
Enterobacteriaceae	2.09 (5.14)	0.33 (0.92)
Muribaculaceae	1.81 (5.93)	0.03 (0.07)
Erysipelotrichaceae	1.53 (6.29)	0 (0.02)
Streptococcaceae	0.74 (1.23)	0.55 (0.98)
Lachnospiraceae	3.64 (6.57)	0.44 (1.35)
Neisseriaceae	0.19 (0.28)	1.01 (1.59)
**Genus**		
Corynebacterium	30.52 (31.18)	0.39 (0.28)
Staphylococcus	17.1 (26.85)	0.1 (0.15)
Dolosigranulum	5.65 (18.15)	0.11 (0.19)
Moraxella	0.23 (0.94)	0.12 (0.25)
Anaerococcus	2.87 (5.21)	0.04 (0.07)
Peptoniphilus	4.13 (7.89)	0.02 (0.04)
Finegoldia	2.01 (4.92)	0.02 (0.05)
Ralstonia	0.81 (1.81)	0.06 (0.14)
Lawsonella	2.12 (4.23)	0.03 (0.06)
Enterobacteriaceae;g_Unclassified	1.84 (4.49)	0.0004 (0.001)
Muribaculaceae	1.81 (5.93)	0.0002 (0.0006)
Dubosiella	1.35 (5.56)	0 (0)
Burkholderia-Caballeronia-Paraburkholderia	4.86 (8.57)	0.01 (0.02)
Streptococcus	0.7 (1.24)	0.01 (0.01)

Deep nasal microbiota of rHC (n=17) and PD (n=30) subject. Mean RA % = average number of sequences per taxa, calculated from the total sum of all sequence counts, depicted as a percentage. Microbial taxa (>1%) shown. (SD) = standard deviation.

Supplementary Table 20. 16S rRNA (V4) differential and compositional abundance analysis of the microbial profiles between random non-household (rHC) healthy control and PD subjects, at the genus taxonomic level.

rHC (n=17) vs. PD (n=30)							
**DESeq2**				**ANCOM**			
**Genus**	**Base Mean**	**Log2 FC (PD over rHC)**	**q value**	**Genus**	**Median Percentile Abundances: rHC**	**Median Percentile Abundances: PD**	**W Score**
Moraxella	3619.03	8.049	**8.29E-04**	*Burkholderia-Caballeronia-Paraburkholderia*	1,842.0	1,574.0	**112**
Veillonella	8.06	6.815	**3.90E-03**	**Genera trending towards significance**			
Comamonas	11.23	6.747	**1.46E-02**	*Bacteroides*	46.6	1.0	89
Rothia	7.53	6.626	**7.67E-03**	***Acinetobacter***	1.0	75.2	57
Enhydrobacter	6.59	6.353	**1.46E-02**	*Faecalibacterium*	17.1	1.0	21
Gemella	3.81	5.796	**2.33E-02**	*Enterobacteriaceae Unclassified*	87.2	1.0	14
Acinetobacter	294.49	5.481	**5.07E-04**				
Pseudomonas	28.72	4.109	**2.33E-02**				

Genera differential and compositional abundance microbiota differences using DESeq2 and ANCOM. Base Mean = mean of normalized samples. Log2 FC = Log2 fold change of taxa in PD compared to rHC subjects. Reference the Methods for DeSeq2 filter details. Median percentile abundance (average of the 25th, 50th, 75th percentiles) and analysis W scores determined using ANCOM. One bacterial genus was significantly identified using ANCOM [filter included removing any sample that was less than 2,300 sequences; any genus that was present in < 2 samples; and whose count across all samples was < 10]. DeSeq2 and ANCOM were corrected for multiple testing using the Benjamini-Hochberg method (FDR-P < 0.05: bold/grey). Taxa identified by both DESeq2 and ANCOM are bolded.

Supplementary Table 21. 16S rRNA (V4) mean relative abundances and corresponding significant differential abundance analysis values of the microbial profiles between spousal household (SpHC) healthy control subjects paired with their corresponding household PD (SpPD) subjects.

**Taxonomic Level**	**Mean RA % ± (SpHC)**	**Mean RA % ± (SpPD)**	**p value**
**Family**			
Bacillales Other	0.0008	0.0004	**0.02**
Neisseriaceae	0.0914	1.6210	**0.02**
Staphylococcaceae	11.1175	2.2657	**0.02**
**Genus**			
Bacillales Other Other	0.0432	0.0048	**0.02**
Kocuria	0.0004	0.0395	**0.04**
Staphylococcus	11.0646	2.1961	**0.01**

Mean RA % = average relative abundance represented as a percentage. Differential abundance differences were measured using Wilcoxon signed-rank paired test within SpHC (n = 11) subjects compared to spPD (n = 11) subjects. Significant P-values (P < 0.05: bold/grey).

a

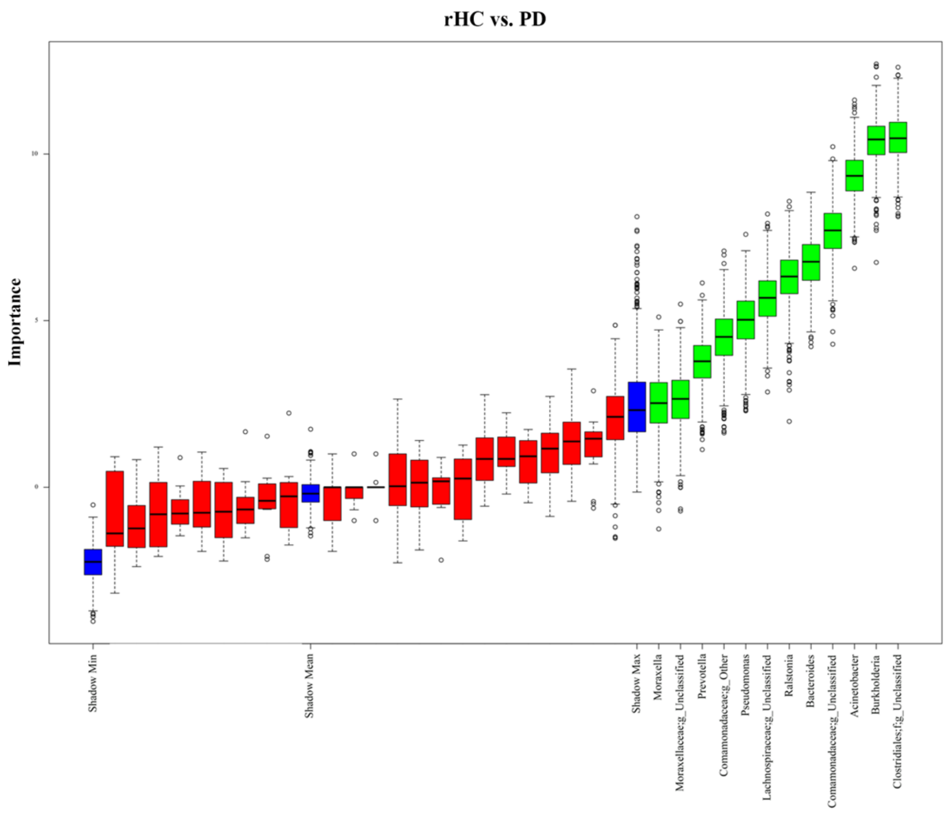

b

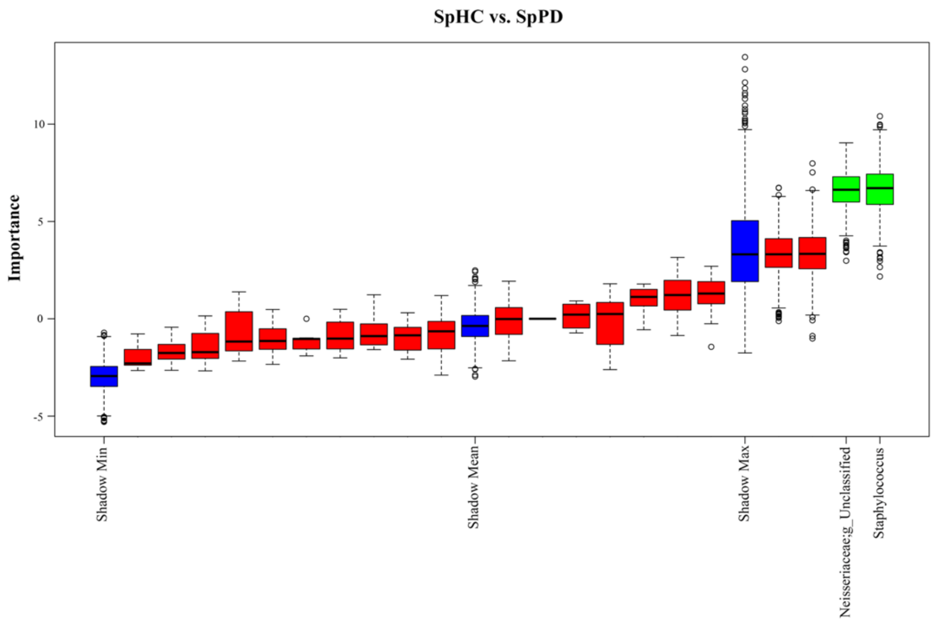

Supplementary Figure 7. Predictive model based on the genus-level differential abundance in PD patients vs. rHC or SpHC vs. SpPD subjects using Boruta for 16S rRNA V4. Green boxes are the bacterial genera that are associated with driving differences between groups were measured using Boruta feature selection algorithm in PD (**A**; n=30) vs. rHC n=17) or SpPD (**B**; n=11) vs. SpHC (n=11). Blue boxes correspond to minimal, average, and maximum Z score of the shadow species, which are shuffled version of real species introduced to Random Forest classifier and act as benchmarks to detect truly predictive species. Red boxes are the bacterial genera that were not associated with driving changes in PD patients were less than the shadow max.

Supplementary Table 22. 16S rRNA (V1-V9) and (V4) dataset comparisons of differential abundance analysis of the microbial profiles between random non-household (rHC) and spousal household (SpHC) healthy controls subjects, at the genus taxonomic level.

**rHC (n=17) vs. SpHC (n=11)**							
**16S rRNA (V1-V9) DeSeq2**				**16S rRNA (V4) DeSeq2**			
**Genus**	**Base Mean**	**Log2 FC (SpHC over rHC)**	**q value**	**Genus**	**Base Mean**	**Log2 FC (SpHC over rHC)**	**q value**
Dermabacter	5.54	5.939	**0.002**	*Comamonas*	2.75	5.895	**0.049**
Delftia	4.84	2.983	**0.006**	*Gemella*	1.71	5.575	**0.049**
Pelomonas	41.64	-2.315	**0.003**	*Lachnospiraceae Unclassified*	166.72	-5.089	**0.012**
Serratia	19.15	-2.622	**0.003**	*Faecalibacterium*	3.46	-6.518	**0.049**
Ralstonia	78.94	-3.052	**2.544E-04**	*Bacteroides*	141.20	-10.670	**1.74E-07**
Lactobacillus	13.84	-3.208	**0.002**	*Burkholderia-Caballeronia-Paraburkholderia*	465.04	-13.174	**4.73E-17**
Blautia	18.58	-3.45	**4.983E-04**				
Bacillus	20.43	-3.559	**4.983E-04**				
Oscillospira	7.17	-3.809	**0.006**				
Burkholderia	290.99	-3.929	**1.640E-14**				
Escherichia	23.67	-4.753	**2.685E-04**				
Paucibacter	8.84	-5.098	**1.111E-05**				
Limnohabitans	7.89	-5.138	**0.001**				
Myroides	4.84	-5.22	**4.997E-04**				
Lentibacillus	1.59	-5.671	**0.003**				
Geothrix	1.98	-5.703	**0.005**				
Curvibacter	11.08	-6.79	**2.119E-05**				
Salinispora	8.6	-7.745	**1.199E-05**				

DESeq2 comparison table depicting the 16S rRNA (V1-V9) and (V4) differential abundant genera between rHC and SpHC subjects. Base Mean = mean of normalized samples. Log2 FC = Log2 fold change of taxa in SpHC compared to rHC subjects. Significant FDR-P values ($q < 0.05$) shown.

Supplementary Table 23. 16S rRNA (V1-V9) and (V4) dataset comparisons of differential abundance analysis of the microbial profiles between random non-household (rHC) and PD subjects, at the genus taxonomic level.

rHC (n=17) vs. PD (n=30)							
**16S rRNA (V1-V9) DeSeq2**				**16S rRNA (V4) DeSeq2**			
**Genus**	**Base Mean**	**Log2 FC (PD over rHC)**	**q value**	**Genus**	**Base Mean**	**Log2 FC (PD over rHC)**	**q value**
Moraxella	7927.57	7.869	**7.840E-10**	***Moraxella***	3619.03	8.049	**8.29E-04**
Methylophaga	5.17	4.908	**0.004**	*Veillonella*	8.06	6.815	**3.90E-03**
Dermabacter	2.72	4.538	**0.001**	*Comamonas*	11.23	6.747	**1.46E-02**
Delftia	8.98	3.283	**1.970E-04**	*Rothia*	7.53	6.626	**7.67E-03**
Ralstonia	1165.2	3.261	**0.004**	*Enhydrobacter*	6.59	6.353	**1.46E-02**
Desulfuromusa	6.47	2.947	**0.008**	*Gemella*	3.81	5.796	**2.33E-02**
Acinetobacter	130.51	1.545	**0.015**	***Acinetobacter***	294.49	5.481	**5.07E-04**
Dietzia	33.18	-1.967	**0.009**	*Pseudomonas*	28.72	4.109	**2.33E-02**
Burkholderia	328.82	-2.046	**0.006**				
Pelomonas	38.18	-2.335	**1.621E-04**				
Listeria	5.09	-2.651	**0.002**				
Serratia	29.92	-2.728	**0.001**				
Bacillus	18.31	-2.812	**0.001**				
Yersinia	38.68	-2.836	**7.078E-06**				
Erythrobacter	1.52	-2.984	**0.003**				
Alkaliphilus	4.49	-2.988	**4.613E-06**				
Lactobacillus	20.32	-3.042	**3.948E-04**				
Lachnospira	3.13	-3.2	**0.003**				
Rhodothermus	0.97	-3.505	**0.005**				
Klebsiella	13.21	-3.701	**0.001**				
Johnsonella	1.25	-4.149	**0.003**				
Paucibacter	7.89	-4.251	**9.790E-08**				
Trabulsiella	17.64	-4.306	**0.001**				
Dechloromonas	0.7	-4.347	**0.002**				
Aminiphilus	0.33	-4.588	**0.002**				
Thalassospira	1.27	-4.913	**0.001**				
Limnohabitans	6.35	-5.091	**5.061E-05**				
Edwardsiella	1.1	-5.465	**0.005**				
Turicibacter	2.04	-5.486	**0.002**				
Gemmatimonas	1.32	-5.574	**0.006**				
Geothrix	1.61	-6.344	**1.701E-05**				
Curvibacter	9.07	-6.581	**1.253E-08**				
DESeq2 comparison table depicting the 16S rRNA (V1-V9) and (V4) differential abundant genera between rHC and PD (n=30) subjects. Taxa identified in both datasets are bolded. Base Mean = mean of normalized samples. Log2 FC = Log2 fold change of taxa in PD compared to rHC subjects. Significant FDR-P values (q < 0.05) shown.							

Supplementary Table 24. 16S rRNA (V1-V9) and (V4) dataset comparisons of differential abundance analysis of the microbial profiles between spousal household (SpHC) healthy control paired to their household PD (SpPD) subjects, at the genus taxonomic level.

**SpHC (n=11) vs. SpPD (n=11)**							
**16S rRNA (V1-V9) DeSeq2**				**16S rRNA (V4) DeSeq2**			
**Genus**	**Mean RA % SpHC**	**Mean RA % SpPD**	**p value**	**Genus**	**Mean RA % SpHC**	**Mean RA % SpPD**	**p value**
Moraxella	0.20	14.84	**0.03**	*Kocuria*	0.00	0.04	**0.04**
Chromobacterium	0.00	0.09	**0.04**	*Bacillales Other Other*	0.04	0.00	**0.02**
Staphylococcus	6.79	1.72	**< 0.01**	***Staphylococcus***	11.06	2.20	**0.01**
Thermodesulfovibrio	0.01	0.00	**0.02**				
Dermacoccus	0.00	0.00	**0.04**				
Wilcoxon signed-rank paired test comparison table depicting the 16S rRNA (V1-V9) and (V4) differential abundant genera between SpHC and SpPD subjects. Taxa identified in both datasets are bolded. Mean RA % = average relative abundance represented as a percentage. Significant P-values ($P < 0.05$) are bolded.							

Supplemental Table 25. Qubit Total DNA Concentrations per sample.

Sample	DNA Concentration: Qubit (ng/ul)
rHC_1	15.4
rHC_2	23.2
rHC_3	18.7
rHC_4	16.8
rHC_5	6.1
rHC_6	14.9
rHC_7	7.7
rHC_8	57.4
rHC_9	15.3
rHC_10	6.2
rHC_11	14.3
rHC_12	8.2
rHC_13	29.0
rHC_14	10.0
rHC_15	18.0
rHC_16	6.7
rHC_17	30.8
SpHC_1	46.3
SpHC_2	39.3
SpHC_3	12.0
SpHC_4	50.0
SpHC_5	100.0
SpHC_6	51.0
SpHC_7	54.0
SpHC_8	20.5
SpHC_9	17.1
SpHC_10	39.6
SpHC_11	31.6
PD_1	100.0
PD_2	51.0
PD_3	52.0
PD_4	44.5
PD_5	46.4
PD_6	52.0
PD_7	30.2
PD_8	58.0
PD_9	59.0
PD_10	34.2
PD_11	11.3
PD_12	8.8
PD_13	59.0
PD_14	100.0
PD_15	100.0
PD_16	44.9
PD_17	100.0

PD_18	59.0
PD_19	20.7
SpPD_PD_20	100.0
SpPD_PD_21	44.4
SpPD_PD_22	14.7
SpPD_PD_23	48.5
SpPD_PD_24	100.0
SpPD_PD_25	59.0
SpPD_PD_26	58.0
SpPD_PD_27	52.0
SpPD_PD_28	55.0
SpPD_PD_29	30.1
SpPD_PD_30	37.4
Samples were verified with fluorometric quantitation (Qubit, Life Technologies, Grand Island, NY, USA). Each sample was eluted with 50uL of DES (DNase/Pyrogen-Free Water).	
